# Unveiling BCL-xL-specific PROTAC efficiency and dissociation pathways using native mass spectrometry

**DOI:** 10.1039/d5sc07400b

**Published:** 2026-02-19

**Authors:** Mohamed I. Gadallah, Kailyn L. Nonhof, Digant Nayak, Peiyi Zhang, Olivia Dioli, Guangrong Zheng, Shaun K. Olsen, Daohong Zhou, Jennifer S. Brodbelt

**Affiliations:** a Department of Chemistry, The University of Texas at Austin Austin TX 78712 USA jbrodbelt@cm.utexas.edu; b Department of Pharmaceutical Analytical Chemistry, Faculty of Pharmacy, Assuit University Assuit 71526 Egypt; c Department of Biochemistry and Structural Biology and Greehey Children's Cancer Research Institute, The University of Texas Health Science Center at San Antonio San Antonio TX 78229 USA; d Department of Medicinal Chemistry, College of Pharmacy, University of Florida Gainesville FL 32611 USA

## Abstract

Overexpression of anti-apoptotic proteins such as BCL-xL is a hallmark of various cancers and a major driver of resistance to conventional chemotherapies. While small-molecule BCL-xL inhibitors have shown promising outcomes, their clinical use is hindered by dose-limiting toxicities, especially thrombocytopenia. Proteolysis-targeting chimeras (PROTACs) offer a promising alternative by promoting selective degradation of target proteins *via* the ubiquitin-proteasome system, thereby reducing off-target effects associated with small molecule inhibitors. However, rational design and optimization of PROTACs remain challenging due to the need to balance simultaneous interactions with both an E3 ubiquitin ligase and the target protein. Here we employ native mass spectrometry (MS) as a rapid, label-free platform to screen and characterize the formation and stability of ternary complexes between BCL-xL, VHL E3 ligase complex (VCB), and various targeting PROTACs. Native MS enables direct detection of binary BCL-xL·PROTAC and ternary BCL-xL·PROTAC·VCB complexes and provides semi-quantitative insights into PROTAC affinity and cooperativity with both binding partners. Furthermore, we explore the dissociation pathways of these complexes in the gas phase using collision-induced dissociation (CID) and ultraviolet photodissociation (UVPD), revealing distinct fragmentation and subunit release patterns that reflect the structural organization and gas-phase stability of the complexes. Variable-temperature ESI-MS (vT-ESI) further allows assessment of thermal stabilities of the complexes in solution. Together, our study demonstrates the power of native MS to both screen and mechanistically characterize PROTAC-induced ternary complex formation.

## Abstract 2

Native mass spectrometry captures PROTAC binding and ternary complex formation, and gas-phase activation (CID, UVPD) and variable-temperature ESI methods delineate the stabilities and stepwise disassembly of PROTAC-induced ternary complexes.

## Graphical abstract

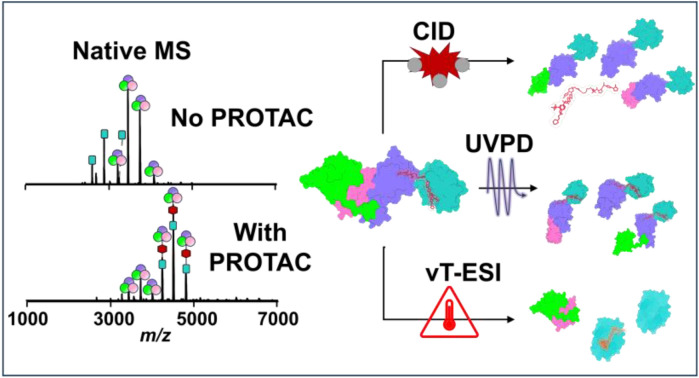

## Introduction

Apoptosis is a highly regulated mechanism of programmed cell death that enables organisms to eliminate unwanted, damaged, or potentially harmful cells in a controlled manner.^1,2^ Unlike necrosis, apoptosis proceeds without triggering an inflammatory response, thereby preserving tissue structure and function.^3^ Dysregulation of apoptotic pathways has been linked to a wide range of diseases, contributing to cancer^4,5^ and autoimmune disorders,^6^ for which insufficient apoptosis allows abnormal cells to survive and proliferate.^6^ Alternatively, excessive apoptosis leads to tissue damage commonly seen in neurodegenerative diseases.^7–9^ A central component of the intrinsic apoptotic pathway is the B-cell lymphoma 2 (BCL-2) protein family, which integrates cellular stress signals to control mitochondrial outer membrane permeabilization, a decisive step that commits cells to apoptosis.^10^ In general, members of the BCL-2 family proteins are broadly categorized into two opposing functional classes. Anti-apoptotic proteins (*e.g.*, BCL-2 and BCL-xL) maintain mitochondrial integrity and support cell survival, whereas pro-apoptotic proteins (*e.g.*, BAX and BAK) promote apoptosis through distinct mechanisms.^10,11^ Despite their functional divergence, BCL-2 family proteins share a conserved BCL-2 homology domain (BH1–BH4) that mediates protein–protein interactions. Anti-apoptotic proteins typically contain all four BH domains, whereas pro-apoptotic effectors possess BH1 to BH3 domains or only BH3. Structurally, these proteins adopt a conserved globular fold composed of eight α-helices (Fig. S1A) connected by flexible loops.^11–13^

Cancer cells often upregulate anti-apoptotic proteins, such as BCL-2 and BCL-xL, to evade apoptosis, maintain uncontrolled proliferation, and disrupt the normal balance between cell survival and programmed cell death.^14,15^ By enhancing the expression of these survival-promoting proteins, cancer cells gain a significant advantage against traditional chemotherapies which ultimately contributes to disease progression and treatment resistance.^4^ Consequently, these anti-apoptotic proteins have emerged as validated targets in cancer therapy.^16,17^ Given the shared structural features of BCL-2 and BCL-xL, dual inhibitors that target both proteins, such as Navitoclax, have been developed.^18,19^ However, clinical use of such agents is limited by their on-target and dose-limiting toxicities, most notably thrombocytopenia, due to the dependence of platelets on BCL-xL for survival.^20^ To overcome these obstacles, BCL-2-selective inhibitors like Venetoclax have been introduced with great success for treatment of hematologic malignancies dependent solely on BCL-2.^21,22^ Nevertheless, cancers that overexpress BCL-xL or co-express both BCL-2 and BCL-x, such as solid tumors and certain lymphomas, remain unresponsive to these inhibitors.^23,24^ An emerging strategy to circumvent these limitations focuses on the use of proteolysis-targeting chimeras (PROTACs).^25–29^ The pharmacological efficacy of these heterobifunctional molecules hinges on their ability to induce the formation of stable ternary complexes between the target protein, the PROTAC, and an E3 ligase, most commonly the VCB complex, which comprises the Von Hippel–Lindau (VHL) substrate recognition protein bound to Elongin B (EloB) and Elongin C (EloC) (Fig. S1B). This assembly brings the target protein into close proximity with the E3 ligase machinery, facilitating ubiquitination of the target protein and subsequent degradation by the proteasome.^27–29^ Unlike small-molecule inhibitors, PROTACs offer a platelet-sparing advantage because platelets express minimal levels of VHL, thereby restricting BCL-xL degradation in these cells and reducing the risk of thrombocytopenia.^25,26^

Characterization of PROTACs, particularly to decipher their binding affinities and stabilities of the key ternary complexes necessary to initiate protein degradation, is essential for advancing new PROTAC-based therapeutics. In this context, native mass spectrometry (MS) has emerged as a powerful alternative and complementary technique for studying protein–protein and protein–ligand interactions, including PROTAC-induced ternary complexes.^30–34^ Unlike conventional denaturing MS, native MS preserves the folded conformations of proteins by ionizing them from aqueous solutions containing volatile, MS-compatible salts such as ammonium acetate. These conditions typically maintain near-physiological pH and ionic strength to minimize the disruption of non-covalent interactions while allowing direct transfer of intact biomolecular complexes into the gas phase by electrospray ionization (ESI).^35–37^ Native MS has been successfully used to study model PROTACs, such as AT1, MZ1, and GNE-987, in complex with Brd4 bromodomains, offering valuable insights into their affinities, complex stoichiometries and cooperativity.^30,31^ Furthermore, gas-phase ion activation methods like collision-induced dissociation (CID) and electron capture dissociation (ECD), have been applied to characterize the topology of PROTAC-induced ternary complexes *via* MS/MS strategies.^32^ Additionally, previous ESI-MS studies have employed organic solvent-induced denaturation, *e.g.*, high percentages of acetonitrile, to investigate the chemical dissociation of PROTAC-induced ternary complexes.^34^ However, this latter approach can disrupt protein structure in a nonspecific manner, making it difficult to distinguish the impact of thermodynamic destabilization from solvent-induced unfolding effects.

Coupling native MS with ultraviolet photodissociation (UVPD) has been utilized to study the dissociation of multimeric protein complexes while preserving native-like interactions.^38^ Unlike collision-based methods such as CID, which often preferentially cleave non-covalent interactions and trigger unfolding prior to subunit ejection, UVPD causes rapid deposition of internal energy without structural rearrangement, offering direct details about subunit architecture.^39,40^ Additionally, variable-temperature electrospray ionization (vT-ESI) combined with native MS enables the study of protein unfolding transitions^41–43^ and stabilities of molecules in solution based on their ion profiles in the mass spectra.^44–48^ The vT-ESI method entails gradually increasing the temperature of solutions containing proteins prior to ESI-MS, allowing real-time monitoring of protein complex dissociation, unfolding and denaturation, and providing information about melting transitions and thermodynamic properties.

In this study, we leverage native MS to assess the formation, specificity, and stability of BCL-xL-targeting PROTAC-induced ternary complexes. We extend the structural characterization of these assemblies by combining complementary ion activation techniques, including CID and UVPD, to evaluate stability, architecture and disassembly pathways of various BCL-xL·PROTAC·VCB complexes in the gas phase. In addition, we employ vT-ESI-MS as an innovative approach for probing in-solution stabilities of BCL-xL PROTAC-induced ternary complexes. Together, these strategies provide deeper insights into PROTAC–protein interactions and support evaluation of newly designed potent and selective degraders.

## Materials and methods

### Materials and reagents

Ammonium acetate, LC-MS-grade water, acetonitrile, methanol, dimethyl sulfoxide, and formic acid were purchased from Sigma-Aldrich (St. Louis, MO). The VCB E3 ligase and BCL-xL were expressed in recombinant *E.coli* strains and affinity-purified, as previously described.^49^ Different active PROTAC molecules including DT2216, 753b and PZ32652, as well as negative control PZ32644, were synthesized in-house as previously described.^50^ The high-affinity VHL inhibitor VH032 and the potent VHL ligand VH298 were obtained from MedChemExpress. The identities (Table S1) of the recombinant proteins, BCL-xL and the VCB complex (*i.e.*, VHL·EloB·EloC), were authenticated using a combination of native MS (Fig. S1C and D), SDS-PAGE (Fig. S1E), and denaturing MS (Fig. S2 and S3). The identity of the PROTACs were also confirmed by mass spectrometry (Fig. S4), and the corresponding structures are shown in Table S2. Other experimental details are included in SI.

### Native mass spectrometry

For native MS, proteins were first desalted and buffer exchanged into 100 mM ammonium acetate using Micro Bio-Spin *P*6 gel columns (Bio-Rad Laboratories Inc.; Hercules, CA). For most experiments, solutions contained one or more of the following: 5 µM BCL-xL, 5 µM PROTAC, and 5 µM VCB complex. DMSO is typically used to prepare concentrated PROTAC stock solutions because it provides excellent solubility and long-term stability, which is standard practice for most PROTAC compounds. However, due to the common occurrence of DMSO-induced charge reduction during native MS (Fig. S5), stocks were diluted into methanol prior to electrospray ionization.^51^ Approximately 2 µL of the resulting solutions were loaded into gold/palladium-coated borosilicate static emitters pulled in-house and subjected to nanoelectrospray ionization using an applied voltage of 0.8–1.1 kV. All native MS experiments were performed using positive polarity on a Thermo Fisher Scientific Q Exactive Plus UHMR (Bremen, Germany) mass spectrometer modified with addition of a Coherent 193 nm/500 Hz excimer laser to enable UVPD in the HCD cell, as described previously.^38,52^ The mass spectrometer was operated using a resolution setting of 1563–3125 (defined at *m*/*z* 400). MS and MS/MS spectra were acquired using a scan range of *m*/*z* 350–8000. An in-source trapping voltage ranging from −50 to −100 V was utilized to enhance the desolvation and transmission of ions. For MS/MS experiments, different complexes (based on isolation of multiple charge states or the individual 16+ charge state) were activated using either higher energy collision dissociation (HCD, a type of CID) or UVPD after quadrupole isolation of the corresponding charge-states. Typically, 50 scans were averaged per spectrum. To evaluate temperature-dependent dissociation, a custom-built variable temperature-ESI source was coupled to the mass spectrometer, as described previously.^53^ ESI mass spectra of solutions containing VCB and PROTAC-induced ternary assemblies were recorded across temperature ranges tailored to their thermal stability, 20–55 °C for the VCB complex, which dissociates fully within this temperature range, and 20–70 °C for the ternary BCL-xL·PROTAC·VCB complexes to encompass their greater thermal stability. These measurements were performed using 2 °C increments with 1.5-minute acquisition per temperature step.

### Denaturing mass spectrometry

Denaturing MS was used to assess the structural integrity and purity of the BCL-xL protein and to detect potential truncations or post-translational modifications. All analyses were performed using a Thermo Scientific Orbitrap Fusion Lumos mass spectrometer (San Jose, CA) modified with addition of a Coherent 193 nm/500 Hz excimer laser. BCL-xL was diluted to 5 µM in 50% methanol containing 0.1% formic acid and buffer-exchanged into the same solvent using 7 kDa MWCO Zeba™ Spin desalting columns (Thermo Scientific). The samples were introduced *via* gold/palladium-coated borosilicate static nanoelectrospray emitters by direct infusion (DI), using an applied spray voltage of 1.0–1.2 kV. Structural analysis of the VCB E3 ligase complex was performed by LC-MS using a Dionex UltiMate 3000 RSLCnano LC system (Thermo Fisher Scientific) in trap-and-elute configuration. Chromatographic separation was achieved using a 3 cm × 100 µm I.D. trapping column and a 20 cm × 75 µm I.D. analytical column, both packed in-house with PLRP-S material (5 µm particle size, 1000 Å pore size; Agilent, Santa Clara, CA). The mobile phases were 0.1% formic acid in water (A) and 0.1% formic acid in acetonitrile (B), delivered at a flow rate of 300 nL min^−1^ using a 90-minute gradient. MS acquisition was performed in positive ion mode over an *m*/*z* range of 400–2,000, using a spray voltage of 1.8 kV. The Orbitrap mass spectrometer was operated at a resolution of 120 000 (at *m*/*z* 200), with 4 microscans, an AGC target of 1 × 10^6^, and a maximum injection time of 100 ms.

### Data analysis

The mass spectra obtained under native conditions using CID (*via* HCD), UVPD, and vT-ESI experiments were deconvoluted using UniDec.^54^ The default parameters established in UniDec were used for spectral deconvolution with some modification, with suitable adjustments made to the mass range (10–75 kDa), charge range (1–30), peak detection threshold (0.05–0.1), and peak detection range (100–500 Da). The mass was sampled every 1 Da. The peaks were fitted using a Gaussian function, and the charge smooth width was 1. The resulting deconvoluted masses for all species are summarized in Table S3. ESIprot was also utilized in conjunction with UniDec to determine the mass and charge states of the different PROTAC ternary complexes.^55^

## Results and discussion

### Initial PROTAC engagement occurs through BCL-xL prior to VCB recruitment

**Fig. 1:**
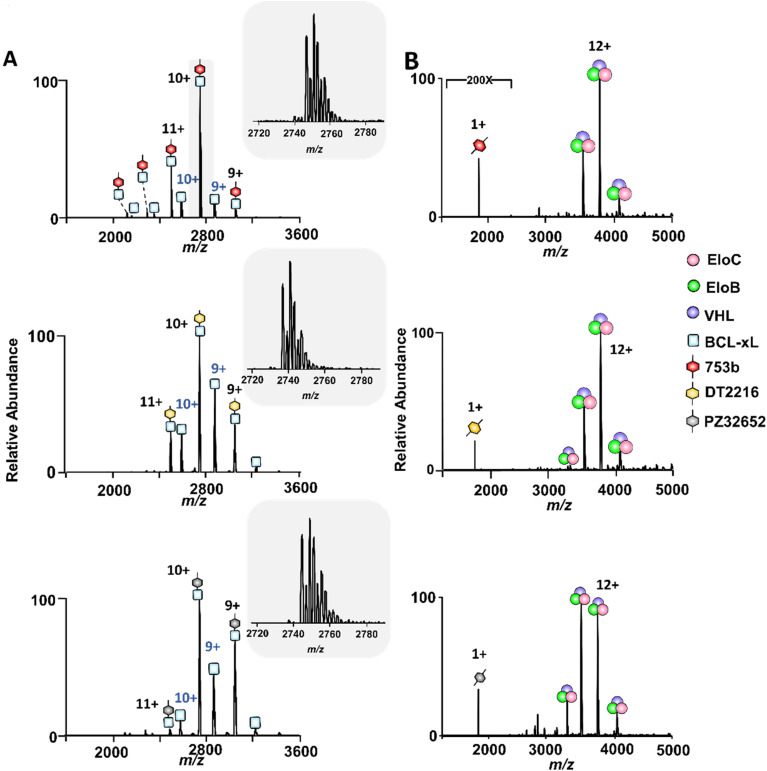
Interactions of PROTACs with BCL-xL or VCB assessed by native MS. Native mass spectra obtained for solutions containing 10 µM of (A) BCL-xL or (B) VCB E3 ligase complex, after incubation with 10 µM PROTAC 753b (top panel), DT2216 (middle panel) or PZ32652 (bottom panel). For the solutions containing a PROTAC and BCL-xL, distinct mass shifts corresponding to the formation of abundant binary BCL-xL·PROTAC complexes are observed with charge state distributions centered around 10+. Expanded views of the 10+ charge state of the BCL-xL·PROTAC complexes show the presence of sodium adducts. No complexes are detected between any PROTAC and the VCB ligase complex.

To probe the interaction hierarchy that governs ternary complex assembly, we first assessed the initial binary interactions of the PROTAC candidates with either BCL-xL or VCB E3 ligase complex using native MS. The three PROTACs investigated, DT2216, 753b, and PZ32652, represent a diverse series of BCL-xL binders that recruit the VHL E3 ligase. DT2216 selectively targets BCL-xL and achieves potent antitumor efficacy while sparing platelets due to minimal expression of VHL in platelets.^25^ 753b is a next-generation analog optimized for improved solubility and cell permeability, and has been shown to induce dual BCL-xL/BCL-2 degradation *in vivo*, thereby broadening therapeutic potential relative to DT2216.^26^ PZ32652, a diastereomeric isomer of 753b, with weaker BCL-xL binding,^50^ serves here as a stereochemical control to assess how chirality and linker orientation influence ternary-complex formation and stability. When incubated with equimolar amounts of either the dual BCL-xL/BCL-2 PROTAC 753b^26^ or the BCL-xL selective PROTAC DT2216 or PZ32652,^25^ abundant binary (BCL-xL·PROTAC) complexes were detected in the ESI mass spectra, with well-resolved charge state distributions centered around the 10+ charge state (Fig. 1A). These mass spectra confirm that all three PROTACs efficiently recognize and engage BCL-xL in a stable manner. The slightly higher signal intensity observed for the BCL-xL·753b complexes relative to the BCL-xL·DT2216 complexes is qualitatively consistent with previously reported *K*_i_ values, in which 753b binds BCL-xL more tightly (1.31 nM) than DT2216 (3.96 nM).^26^ We additionally performed a native MS direct competition experiment in which BCL-xL was incubated simultaneously with equimolar 753b and DT2216; in this case, BCL-xL·753b complexes predominated, supporting the higher relative affinity of 753b (Fig. S6).

In contrast, neither 753b nor DT2216 nor PZ32652 exhibited detectable binding to VCB (*i.e.* no complexes detected) under the same conditions (Fig. 1B). This lack of interaction indicates relatively weak intrinsic affinity between the VHL-binding moiety of the PROTACs and the VCB complex alone, resulting in complexes that are either unstable or below the detection threshold of the mass spectrometer. This was further confirmed by fluorescence polarization (FP) assays, which showed that DT2216 binds VCB only very weakly (IC_50_ = 10.6 µM), in contrast to the high-affinity canonical VHL inhibitor VH032 (IC_50_ = 0.386 µM), a well-established VHL ligand commonly used as a reference for strong VCB engagement (Fig. S7A). Consistent with these results, native MS readily detected abundant binary complexes for high-affinity VHL ligands (VH032 and VH298) for solutions containing 10 µM VCB and 10 µM ligand (Fig. S7B–D). These combined FP and native MS data confirm that the absence of detectable PROTAC·VCB complexes arises from intrinsically weak binary affinities.

### Screening of PROTAC induced ternary complex formation through native MS

**Fig. 2:**
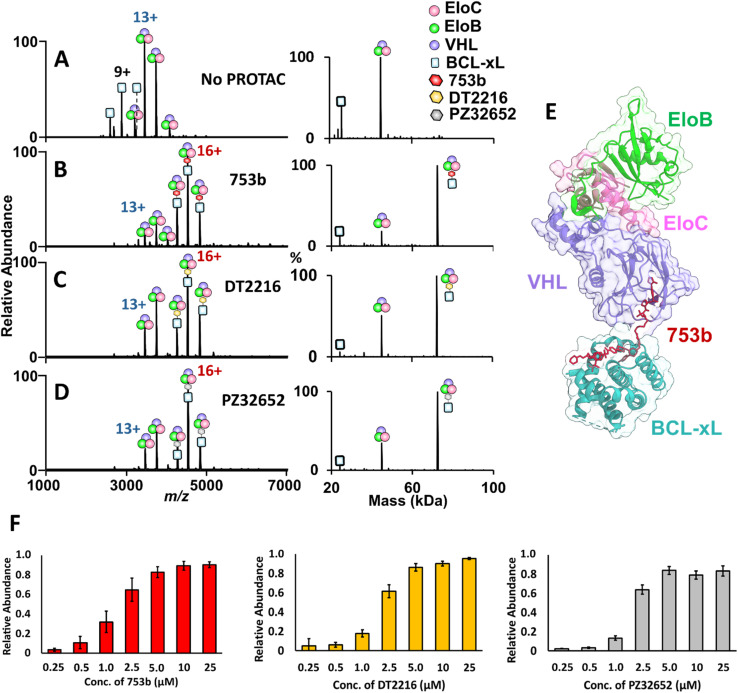
Native MS analysis of PROTAC-induced ternary complex formation with BCL-xL. (A–D) MS1 spectra of solutions containing 5 µM BCL-xL and VCB E3 ligase acquired without and with incubation with 2.5 µM of each PROTAC. Formation of ternary BCL-xL PROTAC·VCB complexes occurred only in the presence of a PROTAC. The corresponding deconvoluted mass spectra are shown on the right. (E) Crystal structure of the ternary complex (8FY0), highlighting BCL-xL (cyan), VHL (purple), EloB (green), and EloC (pink), with PROTAC 753b (red) bridging the interaction.^49^ (F) Bar graphs illustrating relative abundances of ternary BCL-xL·PROTAC·VCB complexes produced upon incubation of 5 µM BCL-xL and 5 µM VCB E3 ligase with increasing concentrations of each PROTAC: 753b (red), DT2216 (gold), and PZ32652 (gray).

A critical step in targeted protein degradation is the formation of ternary complexes involving the E3 ligase complex (VCB), the PROTAC molecule, and the protein of interest (POI).^27–29^ To directly assess ternary complex formation, we utilized native MS, which enables the detection of both binary and ternary assemblies in a label-free and near-native state. This technique offers a distinct advantage over traditional biochemical assays, as it allows direct observation of the assembly and stoichiometries of complexes without the need for protein immobilization or covalent modifications nor the use of secondary detection reagents. In this experiment, equimolar concentrations of BCL-xL and VCB were incubated with 2.5 µM of a BCL-xL PROTAC, either 753b, DT2216, or PZ32652. The latter two PROTACS are less potent BCL-xL degraders that exhibit reduced affinity toward BCL-xL but are still capable of recruiting and engaging the VHL subunit within the VCB E3 ligase complex upon ternary complex formation. In the absence of a PROTAC, only free proteins were detected, confirming no spontaneous formation of ternary complexes (Fig. 2A). Upon addition of a PROTAC that can bind to the VHL subunit within the VCB E3 ligase complex, ions corresponding to fully assembled BCL-xL·PROTAC·VCB complexes emerged (Fig. 2B–D), confirming complex formation in a VHL-dependent manner.

Titration experiments (Fig. S8–S10) allow the visualization of concentration-dependent complex formation and compare the relative binding efficiency of different PROTAC candidates. As shown in Fig. 2C, the resulting dose–response curves revealed small but reproducible differences in the efficiency of ternary complex formation with 753b exhibiting slightly higher binding efficiency based on the production of more abundant complexes at the lower PROTAC concentrations. These differences are modest and consistent with the narrow range of reported dissociation constants (1 nM for 753b and 3–4 nM for DT2216),^26^ suggesting that all three PROTACs engage BCL-xL with similarly high affinity. These titration results provide semi-quantitative insights into the relative binding efficiency of each PROTAC and qualitatively align with previously published cell viability assays showing that 753b exhibits enhanced activity relative to other PROTACs.^26,49^

To further validate these observations, we examined a negative control PROTAC, PZ32644, designed to decouple VHL and BCL-xL interactions. PZ32644 contains an inactive VHL binder (a diastereomeric isomer of the active VHL ligand) and an active BCL-xL binder. PZ32644 shows detectable binding to BCL-xL, as shown by abundant binary complexes (Fig. S11A). However, no interactions between PZ32644 and VCB were observed in the absence of BCL-xL (Fig. S11B), nor were any ternary BCL-xL·PZ32644·VCB complexes detected (Fig. S12). This outcome reinforced the requirement of a functional VHL-engaged PROTAC for bridging the two proteins.

**Scheme 1:**
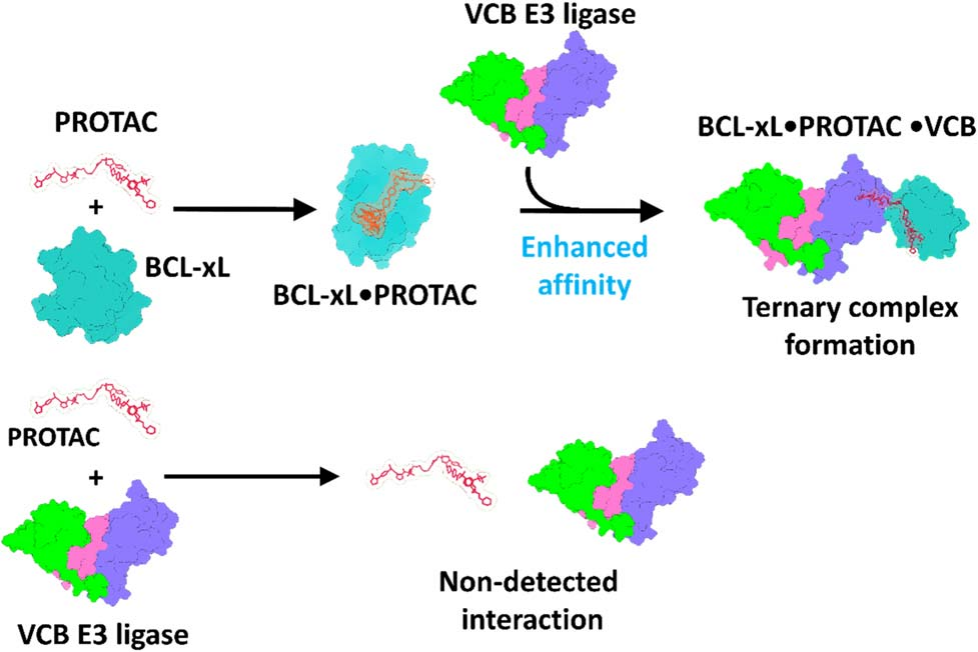
The scheme illustrates that PROTACs show weak or unstable interactions with VCB alone and are not detected by native MS. In contrast, binding to BCL-xL enhances the apparent affinity of the PROTAC toward VCB, enabling formation of stable ternary complexes.

Together, these results suggest the binding affinity of the developed PROTACs to the VCB E3 ligase complex increases substantially in the presence of BCL-xL (Scheme 1). This interaction model indicates that initial binding to BCL-xL enhances subsequent recruitment of VCB, thereby stabilizing the ternary BCL-xL·PROTAC·VCB complexes.

### Probing the disassembly of BCL-xL·PROTAC·VCB complexes using collisional activation

Collisional activation techniques (*i.e.* CID) are widely used strategies to investigate the stabilities^56^ and architectures of protein complexes in the gas phase.^40,57^ These slow-heating methods induce complex dissociation by subjecting ions to multiple low-energy collisions with inert gas molecules, resulting in a gradual buildup of internal energy. For analysis of PROTAC-induced assemblies, we isolated the full charge state envelope of either binary or ternary complexes followed by stepwise application of increasing collision energy for CID. Relative kinetic stabilities in the gas phase are based on measurement of CE_50_ values which correspond to the collision energy at which half of the complexes are dissociated. Binary BCL-xL·PROTAC complexes containing 753b or DT2216 were observed in the 8+ to 10+ charge states, centered at *m*/*z* 3051 and 3047, respectively (Fig. S13). Upon collisional activation, the only prominent dissociation pathway is separation of the BCL-xL protein and PROTAC. These binary BCL-xL·PROTAC complexes exhibited comparatively low stabilities, with CE_50_ values of 13 ± 1 V for the DT2216 complexes and 19.0 ± 0.3 V for the 753b complexes (Fig. S14). The moderately higher CE_50_ value of the binary BCL-xL·753b complexes suggest somewhat greater kinetic stability in the gas phase, which agrees with our previously reported affinity measurements, in which 753b is a more potent BCL-xL PROTAC (*K*_i_ = 1.31 nM) than DT2216 (*K*_i_ = 3.96 nM)^26^ in solution.

**Fig. 3:**
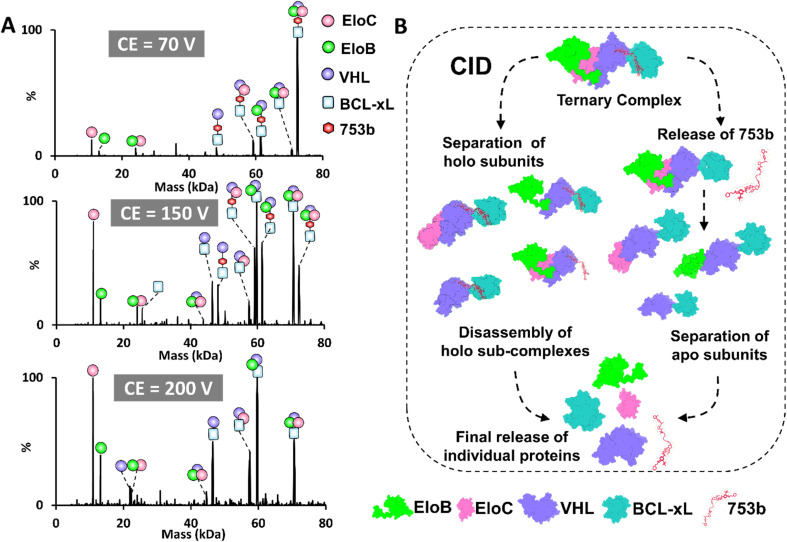
Collision-induced dissociation of ternary BCL-xL·PROTAC·VCB complexes reveals interaction stability and distinct dissociation pathways. (A) Deconvoluted CID mass spectra obtained from ESI-MS/MS analysis of solutions containing 5 µM BCL-xL and 5 µM VCB incubated with 5 µM of 753b. All charge states (14+, 15+, 16+, 17+) were co-isolated and subjected to CID. The ternary complexes dissociate in a stepwise manner by two pathways as the collision energy increases. (B) Schematic representation of the two dissociation pathways observed during CID. The holo-subcomplexes retain PROTAC-mediated interactions between VHL and BCL-xL (left-side), whereas the apo-subcomplexes result from the direct release of 753b (right-side).

The ternary BCL-xL·PROTAC·VCB complexes exhibited charge states ranging from 14+ to 17+, centered at *m*/*z* 4532 for 753b and *m*/*z* 4516 for DT2216 (Fig. S15 and S16). The 14+ to 17+ charge states were co-isolated and subjected to CID, and the use of broadband isolation results in fragmentation that is averaged across the three precursor charge states. Examples of the CID spectra displaying many products in several charge states are shown in Fig. S15 and S16, and representative deconvoluted spectra are shown in Fig. 3A and S17 to simplify the assignment of the products. The major fragmentation pathways upon CID involve the release of VCB subunits (*i.e.* EloC and EloB) (see energy-variable CID profiles in Fig. S18 and S19). The CE_50_ values for the ternary BCL-xL·PROTAC·VCB complexes containing 753b or DT2216 were nearly identical (150 V), and CID provided minimal differentiation between the two PROTACs in terms of their impact on the kinetic stabilities of the complexes. Because CE_50_ values obtained from broadband charge state isolation have the potential to vary because of day-to-day differences in charge-state distributions, we repeated the experiment using individual charge states. Specifically, the 16+ charge state of each ternary complex was isolated and subjected to energy-variable CID (Fig. S20). The dissociation pathways and the extracted CE_50_ values (Fig. S20) were consistent with those obtained from broadband multi-charge state isolation (Fig. S15 and S16) for complexes containing each PROTAC, confirming that the observed dissociation behavior and stability trends are intrinsic to the complexes and not influenced by variations in charge-state distribution.

CID also allowed the subunit dissociation patterns to be probed in more detail.^36,37^ We first examined the dissociation behavior of the VCB E3 ligase complex (EloB·EloC·VHL) using CID. By analyzing VCB alone, we established a reference for the dissociation hierarchy of its subunits, which informs interpretation of the more complex ternary BCL-xL·PROTAC·VCB assemblies. Upon stepwise increase of collision energy, we observed that EloC and EloB are detached from EloB·EloC·VHL at lower energies, while VHL remains associated with either EloC or EloB (see energy-variable CID trends in Fig. S21 and S22). These results suggest that EloC and EloB exhibit weaker gas-phase binding interactions to each other relative to VHL, indicating that VHL maintains more persistent sub-unit interactions upon collisional activation within the VCB E3 ligase complex.

This subunit hierarchy mirrors the dissociation patterns seen for the ternary BCL-xL·PROTAC·VCB complexes, for which VHL remains bound to the PROTAC and target protein, while EloB and EloC are released (Fig. 3A). Specifically, two possible dissociation pathways were detected for both 753b and DT2216 ternary complexes; the first one entails production of holo-subcomplexes in which EloB and EloC are detached first (Fig. 3B left side of panel, Fig. S17E left side of panel), leaving BCL-xL·VHL complexes that retain the PROTAC. This holo subcomplex dissociation pathway confirms that EloB and EloC contribute less to overall complex stability compared to PROTAC-mediated interactions with both VHL and BCL-xL that form the core of the complex. The second dissociation pathway encompasses initial detachment of the PROTAC from BCL-xL·PROTAC·VCB and the detection of intact BCL-xL·VCB binary complexes. Subsequently, EloB and EloC detach, leading to the formation of apo BCL-xL·VHL complexes (Fig. 3B, right side of panel, Fig. S17E, right side of panel). This apo subcomplex dissociation route is an indication of protein unfolding induced through collisional activation.^40^ In fact, it is known that energy accumulation during collisional activation may lead to charge relocalization and cleavage of the weakest bonds (typically non-covalent interactions), causing conformational disruption and detachment of subunits of multimeric complexes.^40^ The stepwise energy deposition of CID can cause structural rearrangement and subunit unfolding prior to dissociation, obscuring analysis of native interaction topologies.^40^ Previous studies examining PROTAC ternary complexes,^32^ such as MZ1 bound to VCB and BRD4 or FKBP12 with specifically designed PROTACs,^34^ have similarly shown that collisional activation preferentially ejects the PROTAC, despite it bridging the structural core of the complex. These limitations underscore the challenges of interpreting ternary complex topological features using CID alone and highlight the need for complementary activation techniques such as UVPD^38–40^ or surface induced dissociation (SID).^58^ SID has been the most popular method for preserving native-like quaternary structures, as the single, high-energy surface collision minimizes gas-phase unfolding and reliably produces subcomplex topologies that reflect the solution assemblies. Similarly, UVPD, when implemented with appropriate energy, can generate comparable subunit hierarchy while providing backbone fragmentation without extensive unfolding, offering complementary insights into both subunit connectivity and sequence features.

### Characterization of BCL-xL·PROTAC·VCB complexes using UVPD

**Fig. 4:**
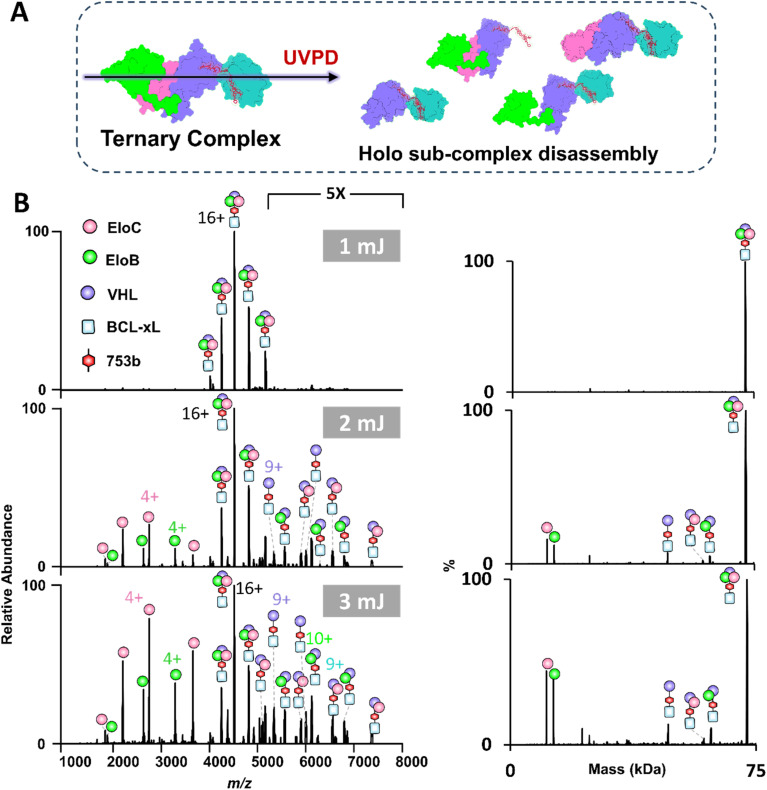
UVPD of the ternary BCL-xL·753b·VCB complexes. (A) Scheme showing the disassembly of ternary complexes by UVPD. (B) UVPD mass spectra of all complexes in the *m*/*z* 4000–5000 range (14+ to 18+ charge states) generated for a solution containing 5 µM BCL-xL and 5 µM VCB incubated with 5 µM PROTAC 753b. UVPD was performed using a single laser pulse, either 1, 2 or 3 mJ. Deconvoluted UVPD mass spectra are shown on the right, confirming the preferential loss of EloB and EloC. The PROTAC 753b remains bound to BCL-xL and VHL, suggesting that UVPD leads to efficient subunit dissociation with minimal disruption of the PROTAC-mediated interactions.

As shown in the previous section, while BCL-xL and VCB proteins do not interact under native conditions without a PROTAC, CID of the BCL-xL·PROTAC·VCB ternary complexes unexpectedly resulted in the formation of binary BCL-xL·VCB complexes in conjunction with the release of the PROTAC. This outcome suggests that the slow energy deposition characteristic of collisional activation promotes partial unfolding of protein subunits, which may expose non-native interfaces and lead to the formation of artificial assemblies not representative of biologically relevant states. To address this concern and obtain meaningful structural insights while preserving native topology, we employed UVPD as a complementary method to characterize both VCB E3 ligase and PROTAC induced tertiary complexes. UVPD is known for higher energy deposition in a faster process that minimizes unfolding prior to disassembly of multimeric assemblies.^38–40^ For activation of VCB E3 ligase (11+ to 14+), UVPD primarily resulted in release of the peripheral EloB and EloC subunits (Fig. S23). Next UVPD was used to characterize the ternary BCL-xL·753b·VCB and BCL-xL·DT2216·VCB complexes (Fig. 4 and S24). To further simplify spectral interpretation and enable a direct, charge-state–matched comparison with the CID data acquired for the 16+ charge state, we also performed UVPD on the isolated 16^+^ charge state of each ternary complex (Fig. S25), which yielded cleaner fragment ion distributions and preserved the same subunit-ejection hierarchy observed in the broadband UVPD experiments. In contrast to CID, UVPD consistently produced dominant BCL-xL·PROTAC·VHL subcomplexes, preserving the core architecture of the ternary complex, while selectively releasing the more weakly bound peripheral subunits, namely EloB and EloC. This dissociation behavior highlights the ability of UVPD to retain inter-subunit contacts during activation and to provide a more accurate depiction of the quaternary organization. Hence, the combined use of CID and UVPD provides complementary structural perspectives on PROTAC-induced complexes. CID primarily reports on stability and unfolding behavior, whereas UVPD better captures topology, inter-subunit contacts and quaternary organization.

The low-abundance ion peaks observed at higher UVPD laser energies (Fig. 4B, 3 mJ) correspond to fragment ions generated from backbone cleavages of the protein subunits. These ion peaks are not annotated owing to the high density of the spectrum and because the spectra were acquired using low mass resolution to minimize ion decay within the Orbitrap mass analyzer, creating conditions that are not optimal for resolving and confidently assigning backbone fragment ions. We performed an additional UVPD experiment using a lower trapping gas pressure and higher-resolution setting to enhance identification of these fragment ions. As shown in Fig. S26A, additional low-abundance ions are detected, confirming that the previously unannotated ion peaks arise from backbone fragmentation. However, due to the large size of the complex, the resulting spectra are highly congested and provided limited sequence coverage (Fig. S26B). Comprehensive mapping of these fragments would likely require using ion–ion charge reduction (PTCR) or a higher-capacity mass spectrometer.

### Probing the disassembly and stability of different PROTAC ternary complexes under thermal stress using variable temperature-ESI native MS

Although ion activation techniques like CID and UVPD provide insights about dissociation of multimeric complexes in the gas phase, it is important to understand how complexes disassemble in solution. To accomplish this aim, we employed vT-ESI to monitor the thermal dissociation of BCL-xL·753b·VCB and BCL-xL·DT2216·VCB ternary complexes in solution. This method tracks the relative abundances of ternary complexes and their dissociation products as a function of temperature as the protein solution is heated and electrosprayed, providing critical information about the thermal stability and disassembly of the complexes. Initially, we examined the thermal-induced dissociation of the VCB E3 ligase complex alone over a solution temperature range of 20–55 °C, the first step in understanding the disassembly of higher order ternary complexes. As the solution temperature is increased, the VCB complexes decay and binary complexes (EloC·EloB, EloB·VHL, and EloC·VHL) are detected (Fig. S27), and EloC·EloB shows the greatest stability at higher temperatures.

**Fig. 5:**
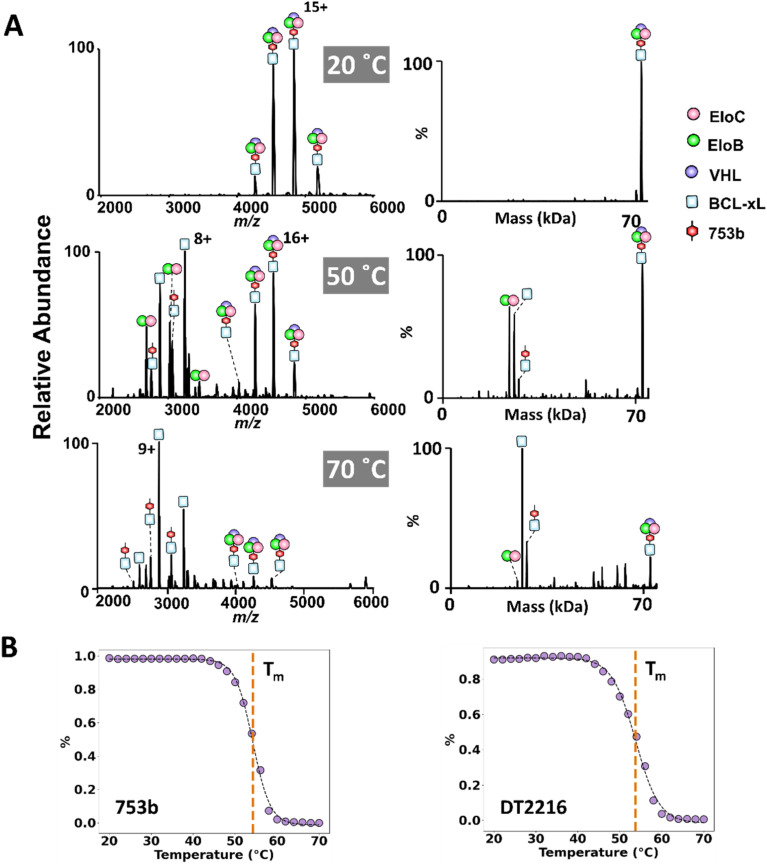
Probing the disassembly and stability of PROTAC ternary complexes using variable temperature-ESI-MS. Solutions containing 5 µM BCL-xL and 5 µM VCB in 100 mM ammonium acetate incubated with 5 µM of 753b or DT2216 were gradually heated using a variable temperature-ESI source over a temperature range of 20 °C to 70 °C. (A) ESI-mass spectra were acquired at multiple temperatures. The deconvoluted mass spectra are shown on the right. (B) Melting curves for the solutions containing BCL-xL and VCB with 753b (left) or with DT2216 (right) illustrate the temperature-dependent dissociation of the ternary complexes. The melting curves were generated by fitting the relative abundances of the ternary BCL-xL·PROTAC·VCB complexes as a function of temperature using a sigmoidal model, yielding melting temperatures of 54.2 + 0.8 °C for 753b and 53.8 + 0.8 °C for DT2216 (not a significant difference).

The variable temperature experiments were then repeated for solutions containing BCL-xL, VCB, and either DT2216 or 753b. Abundant ternary BCL-xL·PROTAC·VCB complexes were observed at low temperature (20 °C), with minimal dissociation (Fig. 5A). As the solution temperature was increased, more dissociation was evident, resulting in the appearance of EloC·EloB, free BCL-xL, and binary BCL-xL·PROTAC complexes (Fig. 5A and S28). Notably, no binary BCL-xL·VCB complexes were detected at any temperature, indicating that this species—previously observed by CID but not UVPD—does not form under native solution conditions and likely arises as an artifact of gas-phase unfolding and rearrangement.

Melting curves derived from the abundances of the ternary BCL-xL·PROTAC·VCB complexes as a function of solution temperature revealed melting temperatures (*T*_m_) of 54.2 + 0.8 °C for 753b and 53.8 + 0.8 °C for DT2216 (Fig. 5B). These similar *T*_m_ values indicate comparable thermal stabilities of the ternary complexes containing each of the two PROTACS (Fig. S29). Notably, the preservation of ternary complexes at physiological temperatures supports the stability of both PROTAC-mediated assemblies under biologically relevant conditions. These data underscore the utility of vT-ESI in evaluating thermal stabilities of complexes.

## Conclusion

Our comparative native MS analysis revealed that distinct PROTACs differ in their ability to form stable, cooperative complexes with both BCL-xL and the VHL E3 ligase. Native MS of the binary assemblies confirmed that all three PROTACs, DT2216, 753b, and PZ32652, bind BCL-xL efficiently, with 753b forming the highest proportion of binary complexes, followed by PZ32652 and then DT2216. Upon addition of the VCB ligase, all three PROTACs promoted formation of ternary BCL-xL·PROTAC·VCB assemblies, with 753b displaying a slightly enhanced, though not statistically significant, degree of cooperativity. These results qualitatively align with their cellular degradation profiles,^26,49^ wherein 753b drives more extensive BCL-xL degradation and stronger apoptotic responses than DT2216. Together, these findings demonstrate that native MS can differentiate variations in affinity and cooperativity among structurally related PROTACs, which may provide mechanistic context for differences observed in cellular degradation outcomes. In addition, our results highlight the complementary strengths and limitations of CID, UVPD, and vT-ESI for evaluation of PROTAC ternary complexes. CID provides compositional information and reveals dissociation hierarchies, but can induce gas-phase unfolding due to its slow, stepwise energy deposition. This effect may cause non-native dissociation pathways, including premature PROTAC ejection, limiting the structural insight of CID alone. In contrast, UVPD offers fast higher energy deposition that causes disassembly into holo-subcomplexes without evidence for re-organization. As such, UVPD produces subunit-ejection patterns that more accurately reflect native interface topology of ternary complexes. vT-ESI probes in-solution thermal stability and unfolding of PROTAC-induced complexes, revealing solution stability trends and cooperativity that cannot be inferred from activation of gas-phase complexes.

## Author contributions

Conceptualization: Mohamed I. Gadallah; Jennifer S. Brodbelt; methodology: Mohamed I. Gadallah; Kailyn L. Nonhof; Digant Nayak; Peiyi Zhang; writing – original draft: Mohamed I. Gadallah; writing – review and editing: Mohamed I. Gadallah; Digant Nayak; Peiyi Zhang; Olivia Dioli; Guangrong Zheng; Shaun K. Olsen; Daohong Zhou; Jennifer S. Brodbelt; supervision: Guangrong Zheng; Shaun K. Olsen; Daohong Zhou, Jennifer S. Brodbelt; funding acquisition: Guangrong Zheng; Shaun K. Olsen; Daohong Zhou; Jennifer S. Brodbelt.

## Conflicts of interest

P. Z., G. Z., and D. Z. are inventors of the patents and pending patent applications for use of BCL-xL PROTACs as senolytic and antitumor agents. G. Z., and D. Z. are co-founders of and have equity in Dialectic Therapeutics, which develops BCL-xL/2 PROTACs to treat cancer. All other authors declare no competing financial interest.

## Supplementary Material

SC-017-D5SC07400B-s001

## Data Availability

All data have been deposited into the jPOST public repository^59^ with the accession number JPST004199. Supplementary information (SI): sequences of all proteins, structures of PROTACs, numerous MS1 spectra showing formation of complexes and competition and titration data, numerous MS/MS spectra and energy-variable breakdown curves, temperature dependent data, and additional experimental details. See DOI: https://doi.org/10.1039/d5sc07400b.
