# Supplementary material for: Unveiling BCL-xL-specific PROTAC efficiency and dissociation pathways using native mass spectrometry
Source: Chem Sci. 2026 Feb 19;17(14):7297–309. doi: 10.1039/d5sc07400b (PMC12917756; doi:10.1039/d5sc07400b)
Supplement: SC-017-D5SC07400B-s001 [file SC-017-D5SC07400B-s001.pdf]

Supplementary Information (SI) for Chemical Science.
This journal is © The Royal Society of Chemistry 2026

Supporting Information

Unveiling BCL-xL specific PROTAC Efficiency and Dissociation Pathways Using Native Mass Spectrometry

Mohamed I. Gadallah[1,2], Kailyn L. Nonhof[1], Digant Nayak[3], Peiyi Zhang[4], Olivia Dioli[1], Guangrong Zheng[4], Shaun K. Olsen[3], Daohong Zhou[3], Jennifer S. Brodbelt[1]*

[1]Department of Chemistry, The University of Texas at Austin, Austin, TX, 78712, United States
[2]Department of Pharmaceutical Analytical Chemistry, Faculty of Pharmacy, Assuit University, Assuit 71526, Egypt
[3]Department of Biochemistry and Structural Biology, University of Texas Health Science Center at San Antonio, San Antonio, TX
[4]Department of Medicinal Chemistry, College of Pharmacy, University of Florida, Gainesville, FL

*Correspondence to: Jennifer S. Brodbelt, jbrodbelt@cm.utexas.edu

Content	Page no.
**Table S1.** Sequences and average masses of BCL-xL proteins and VCB E3 ligase subunits. The amino acids comprising different BCL-xL protein helices are highlighted with different colors,.	S4
**Table S2.** Chemical structures and monoisotopic mass for the studied PROTACs.	S5
**Table S3.** Theoretical and experimental masses of all protein and complexes detected by Native Mass Spectrometry	S6
**Figure S1. Analysis of BCL-xL anti-apoptotic proteins and VCB E3 ubiquitin ligase**. Crystal structure of (A) BCL-xL (PDB: 1R2D) highlighting its helical regions and (B) the VCB complex (PDB: 8B0F) showing the three subunits: VHL (purple), EloB (green), and EloC (pink). MS1 spectra of solutions containing 10 μM of (C) BCL-xL, or (D) VCB E3 ligase after buffer exchange into 100 mM ammonium acetate, confirming the identity and purity of the expressed proteins. (E) SDS-PAGE gels showing the purification of (top) BCL-xL and (bottom) the VHL–EloB–EloC (VBC) complex.	S7
**Figure S2. Analysis of BCL-xL proteins using denaturing conditions**. MS1 spectra of solutions containing 5 μM of BCL-xL in 50% methanol containing 0.1% formic acid analyzed using an Orbitrap Fusion Lumos mass spectrometer confirming the identity and purity of the expressed proteins. The inset shows the corresponding deconvoluted mass spectrum.	S8
**Figure S3. Characterization of the VCB E3 ligase protein complex using denaturing nano-LC-MS.** Extracted ion chromatograms acquired from 30 to 55 minutes showing the separation of individual VCB subunits: (A) Elongin B (13.1 kDa), (B) VHL (20.9 kDa), and (C) Elongin C (10.9 kDa). (D-F) Corresponding mass spectra of the separated subunits acquired at the elution times shown on the left, confirming their identity and purity.	S9
**Figure S4.** MS1 spectra of solutions containing 5 μM of a PROTAC or VHL inhibitor acquired on a Thermo HFX Orbitrap mass spectrometer. Panels A–D show spectra for the four PROTACs: (A) 753b (B) DT2216 (C) PZ32644 and (D) PZ32652. Panels E–F show spectra for the VHL inhibitors: (E) VH298 and (F) VH032 (the latter mainly as a sodium adduct). All spectra were collected in methanol with 0.1% formic acid using a spray voltage of 0.8 kV and a resolution setting of 45,000.	S10
**Figure S5. MS1 spectra of BCL-xL and VCB E3 ligase in different solvent conditions.** MS1 spectra of 10 μM of BCL-xL (left-side) or VCB E3 ligase (right-side) sprayed from 100 mM ammonium acetate (A and D) without or with the addition of (B and E) 10% DMSO or (C and F) 10% MeOH. The addition of 10% DMSO results in a slight shift towards lower charge states.[1] In contrast, the addition of 10% MeOH does not significantly alter the charge state distribution.	S11
**Figure S6. Direct competition experiment demonstrates preferential binding of 753b over DT2216 to BCL-xL.** Native MS spectrum of BCL-xL incubated simultaneously with equimolar 753b and DT2216 (10 μM each). The spectrum shows dominant formation of the BCL-xL•753b binary complexes with lower formation of the BCL-xL•DT2216 complexes, supporting that 753b outcompetes DT2216 for BCL-xL binding under identical conditions.	S12

Content	Page
**Figure S7. Evaluation of VCB binding to PROTAC DT2216 and high-affinity VHL Inhibitors.** (A) Fluorescence polarization (FP) binding curves for DT2216 and VH032 measured against VCB. The low DT2216–VCB affinity explains the absence of detectable binary PROTAC–VCB complexes in the native ESI mass spectra. Native mass spectra acquired under identical conditions as used for the other PROTAC experiments for solutions containing: B) 10 mM VCB alone, C) 10 mM VCB and 10 mM VH032 (IC50 = 185 nM) and D) 10 mM VCB and 10 mM VH298 (IC50 = 90 nM).[2]	S13
**Figure S8. Titration of BCL-xL and VCB with 753b PROTAC monitored using native MS.** (A) ESI mass spectra obtained for solutions containing 5 µM BCL-xL and 5 µM VCB in 100 mM ammonium acetate with various concentrations of 753b: A) 1.25 µM, B) 2.5 µM, C) 5 µM, or D) 10 µM. The corresponding deconvoluted spectra are shown on the right.	S14
**Figure S9. Titration of BCL-xL and VCB with DT2216 PROTAC monitored using native MS.** (A) ESI mass spectra obtained for solutions containing 5 µM BCL-xL and 5 µM VCB in 100 mM ammonium acetate with various concentrations of DT2216: A) 1.25 µM, B) 2.5 µM, C) 5 µM, or D) 10 µM. The corresponding deconvoluted spectra are shown on the right.	S15
**Figure S10. Titration of BCL-xL and VCB with PZ32652 PROTAC using native MS.** (A) ESI mass spectra obtained for solutions containing 5 µM BCL-xL and 5 µM VCB in 100 mM ammonium acetate and various concentrations of PZ32652: A) 1.25 µM, B) 2.5 µM, C) 5 µM, or D) 10 µM. The corresponding deconvoluted spectra are shown on the right.	S16
**Figure S11. Binary interactions of negative control PROTAC PZ32644 with BCL-xL and VCB evaluated by native MS.** ESI mass spectra obtained for solutions containing 10 µM of (A) BCL-xL or (B) VCB E3 ligase complex after incubation with 10 µM PROTAC PZ32644.	S17
**Figure S12. PROTAC PZ32644 does not induce ternary complex formation.** (A) MS1 spectrum of a solution containing 5 µM BCL-xL and 5 µM VCB E3 ligase incubated with 5 µM of a non-binding PROTAC (PZ32644), following buffer exchange into 100 mM ammonium acetate.	S18
**Figure S13. Evaluation of the gas-phase stabilities of binary BCL-xL•PROTAC complexes based on energy-variable CID.** CID mass spectra of BCL-xL•PROTAC complexes (co-isolated 8+ to 11+ charge states) containing (A-D) 753b or (E-H) DT2216 acquired at selected collision voltages.	S19
**Figure S14.** Energy-variable CID curves for +8 to +10 charge state of the binary complexes BCL-xL•753b (red) and BCL-xL•DT2216 (gold) with the CE_{50} values marked with dashed lines.	S20
**Figure S15. Energy-variable CID of ternary BCL-xL•753b•VCB complexes**. CID mass spectra of the BCL-xL•753b•VCB ternary complex (co-isolated 14+ to 17+ charge states) using CID voltages of: (A) 10 V, (B) 70 V, (C) 150 V, and (D) 200 V.	S21
**Figure S16. Energy-variable CID of ternary BCL-xL•DT2216•VCB complexes**. CID mass spectra of the BCL-xL•DT2216•VCB ternary complex (co-isolated 14+ to 17+ charge states) using CID voltages of: (A) 10 V, (B) 70 V, (C) 150 V, and (D) 200 V.	S22
**Figure S17. Collision-induced dissociation of ternary BCL-xL•DT2216•VCB complexes reveals two distinct dissociation pathways. (A-D)** Deconvoluted CID mass spectra obtained from ESI-MS/MS analysis of solutions containing 5 µM BCL-xL and 5 µM VCB incubated with 5 µM of DT2216 (co-isolated 14+ to 17+ charge states) as a function of CID collision energy. The ternary complexes dissociate in a stepwise manner by two pathways as the collision energy increases. (E) Schematic representation of the two dissociation pathways observed during CID.	S23
**Figure S18. Product ion distributions based on energy-variable CID of PROTAC-mediated ternary complexes for** (A) BCL-xL•753b•VCB (co-isolated 14+ to 17+ charge states) and (B) BCL-xL•DT2216•VCB (co-isolated 14+ to 17+ charge states). The resulting subcomplexes and monomeric subunits were tracked as a function of increasing collision energy. The curves were constructed using UniDec deconvolution of each CID spectrum, followed by extraction of the integrated peak areas corresponding to the intact ternary complex and all resolved subcomplexes.	S24
**Figure S19.** Dissociation profiles of the individual ions tracked in **Figure S18**. (A) BCL-xL•753b•VCB (co-isolated 14+ to 17+ charge states) and (B) BCL-xL•DT2216•VCB (co-isolated 14+ to 17+ charge states).	S25
**Figure 20.** Energy-variable CID of the 16+ charge state of (A) BCL-xL·753b·VCB and (B) BCL-xL·DT2216·VCB ternary complexes generated from solutions containing 5 µM BCL-xL and 5 µM VCB incubated with 5 µM of the respective PROTAC**.** (C) Energy-variable CID breakdown graph displaying the abundance of the precursor as the collision energy is varied. Both ternary complexes exhibited comparable dissociation behavior and kinetic stabilities.	S26

Content	Page no.
**Figure S21. Dissociation behavior of the VCB E3 ligase complexes as a function of CID collision voltage. CID** mass spectra of the VCB complexes (co-isolated 11+ to 14+ charge states) acquired using different CID voltages, revealing the progressive release of subunits EloB and EloC. (D) The relative abundances of individual subunits and subcomplexes are plotted as a function of CID collision voltage, demonstrating stepwise dissociation.	S27
**Figure S22**. Dissociation profiles of the individual ions tracked in **Figure S21 for** VCB (VHL•EloC•EloB) (co-isolated 11+ to 14+ charge states).	S28
**Figure S23. Dissociation behavior of the VCB E3 ligase complexes using UVPD.** UVPD mass spectra of the VCB complexes (co-isolated 11+ to 14+ charge states) acquired using different UVPD energy (A) 1 mJ, (B) 2 mJ and (C) 3 mJ, revealing the progressive release of subunits EloB and EloC.	S29
**Figure S24. UVPD mass spectra of the ternary BCL-xL•DT2216•VCB complexes.** (A-C) A single 1–3 mJ laser pulse was applied after co-isolation of ions in the *m/z* 4,000–5,000 range encompassing the 14+ to 18+ charge states. (D-F) The deconvoluted mass spectra are shown on the right, confirming the preferential loss of EloB and EloC, while the DT2216 remains bound to the rest of the complex, suggesting that UVPD leads to direct subunit dissociation with minimal disruption of the PROTAC-mediated interactions.	S30
**Figure S25.** Isolation and UVPD of the ternary BCL-xL•PROTAC•VCB complexes (16+) for complexes generated from a solution containing 5 μM BCL-xL and 5 μM VCB incubated with 5 μM PROTAC (A-D) 753b and (E-F) DT2216. UVPD was performed using a single laser pulse, 1 - 3 mJ.	S31
**Figure S26. UVPD fragmentation of the BCL-xL•753b•VCB ternary complex in the 16+ charge state.** (A) UVPD mass spectrum using single pulse and 3 mJ laser energy, 240 K resolution, and trapping gas setting = 2. The high-resolution, low-pressure conditions enhance detection of backbone-type fragment ions but also lead to a highly congested spectrum due to the large size of the ternary complex. (B) Sequence-coverage maps highlight the observed a, b, c, x, y, and z ions, confirming that the low-abundance peaks originate from backbone fragmentation rather than subcomplex dissociation.	S32
**Figure S27. Thermal-induced dissociation of VCB complex using variable temperature-ESI-MS.** The experiment was conducted using a solution containing 5 μM VCB in 100 mM ammonium acetate. The solution was gradually heated over a temperature range of 20°C to 55°C. Mass spectra were acquired at multiple temperature points including A) 20°C, B) 35°C and C) 50°C. (D) The relative abundances of key molecular species are plotted as a function of temperature, showing a stepwise dissociation pattern, with EloC•EloB released first, followed by the sequential dissociation of individual subunits.	S33
**Figure S28. Evaluation of the thermal-induced stability of complexes containing DT2216 using variable temperature ESI-MS.** The solution contained 5 μM BCL-xL, 5 μM VCB and 5 μM DT2216 in 100 mM ammonium acetate. The solution was gradually heated over a temperature range of 20°C to 70°C. Mass spectra were acquired at A) 20°C, B) 50°C, and C) 70°C. Deconvoluted mass spectra are shown on the right, confirming the sequential release of EloC•EloB followed by release of BCL-xL and disassembly of the binary BCL-xL•DT2216 complex.	S34
**Figure S29. Temperature-dependent stability assay of different PROTAC complexes analyzed by variable temperature-ESI-MS.** The relative abundances of key molecular species are plotted as a function of temperature for complexes in solutions containing A) BCL-xL, 753b, and VCB or (B) BCL-xL, DT2216 and VCB. The experiments were conducted using solutions containing 5 μM BCL-xL, 5 μM VCB and 5 μM PROTAC.	S35
Protein expression and synthesis of PROTACs. Fluorescence polarization assay.	S36
Authentication of materials	S37
References	S38

Table S1. Sequences and average masses of BCL-xL proteins and VCB E3 ligase subunits. The amino acids comprising different BCL-xL protein helices are highlighted with different colors,.

Protein	Sequence	Average theoretical mass (Da)	Average experimental mass (Da)
**BCL-xL**	**GSSHHHHHHSSGLVPRG**SHMSQSNRELVVDF LSYKLSQKGYSWSQFSDVEENRTEAPEGTESEM ETPSAINGNPSWHLADSPAVNGATGHSSSLDA REVIPMAAVKQALREAGDEFELRYRRAFSDLTS QLHITPGTAYQSFEQVVNELFRDGVNWGRIVAF FSFGGALCVESVDKEMQVLVSRIAAWMATYLN DHLEPWIQENGGWDTFVELYGNNAAAESRKG QERFNR	25,816	25,816
**VHL**	MGSSHHHHHHSSGLVPRGSMEAGRPRPVLRS VNSREPSQVIFCNRSPRVVLPVWLNFDGEPQP YPTLPPGTGRRIHSYRGHLWLFRDAGTHDGLLV NQTELFVPSLNVDGQPIFANITLPVYTLKERCLQ VVRSLVKPENYRRLDIVRSLYEDLEDHPNVQKDL ERLTQERIAHQRMGD	20,558	20,962
**Elongin C**	MYVKLISSDGHEFIVKREHALTSGTIKAMLSGPG QFAENETNEVNFREIPSHVLSKVCMYFTYKVRY TNSSTEIPEFPIAPEIALELLMAANFLDC	10,832	10,962
**Elongin B**	MDVFLMIRRHKTTIFTDAKESSTVFELKRIVEGIL KRPPDEQRLYKDDQLLDDGKTLGECGFTSQTAR PQAPATVGLAFRADDTFEALCIEPFSSPPELPDV MKPQSGSSANEQAVQ	13,017	13,131

Table S2. Structures and monoisotopic masses of the PROTACs and VHL ligands.

PROTAC	Chemical Structure	Monoisotopic mass (Da)
**753b**	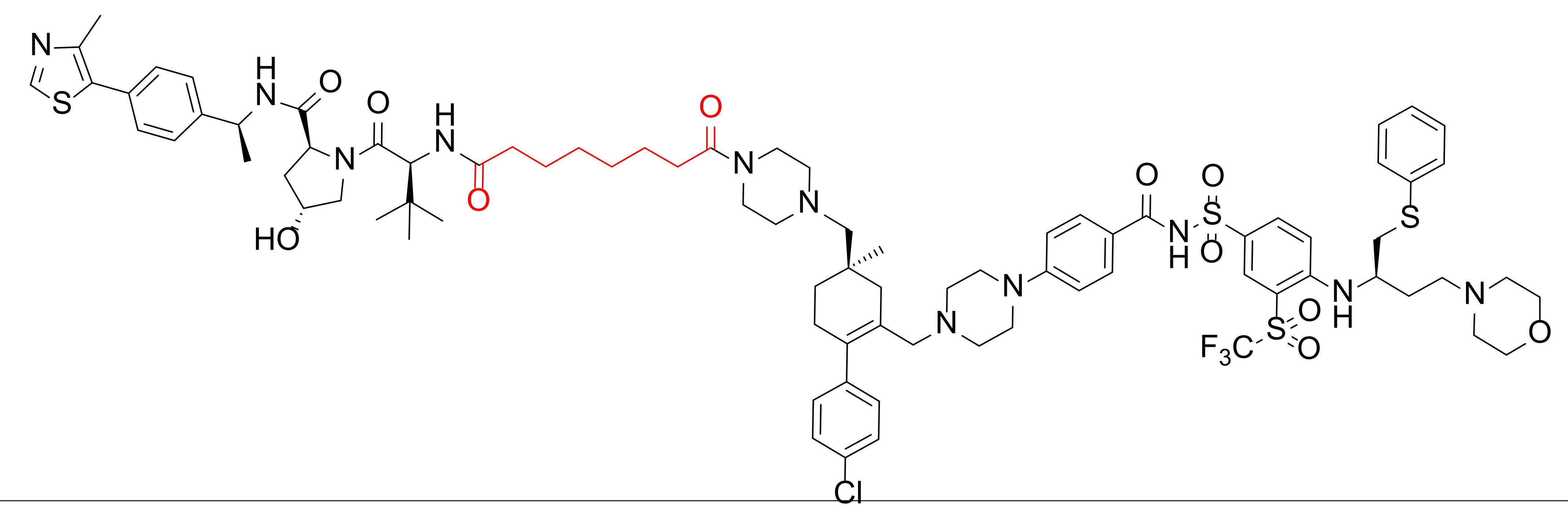	**1639.65**
**DT2216**	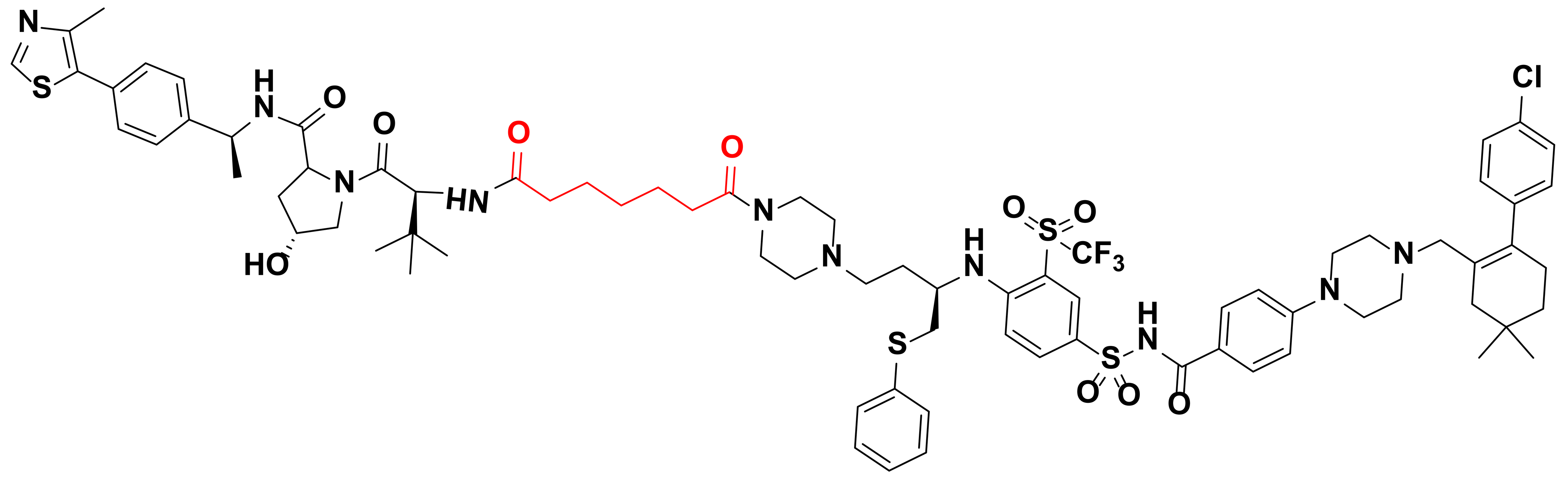	**1540.58**
**PZ32644**	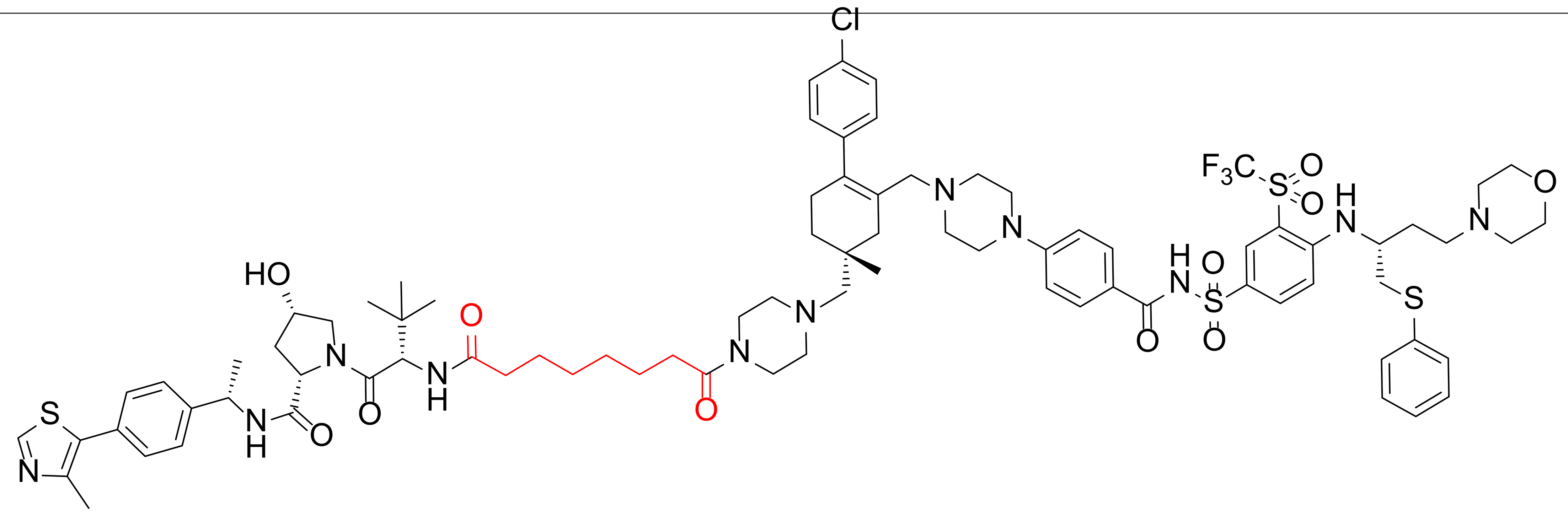	**1639.65**
**PZ32652**	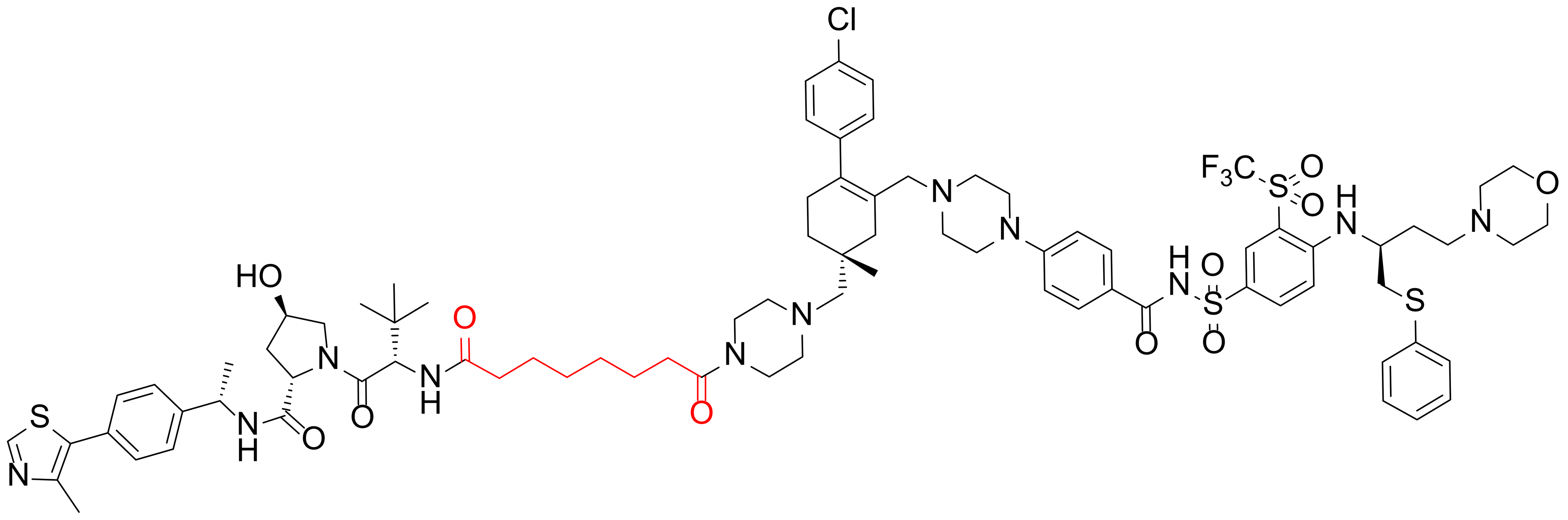	**1639.65**
**VH298 (VHL ligand K_d 80-90 nM)**	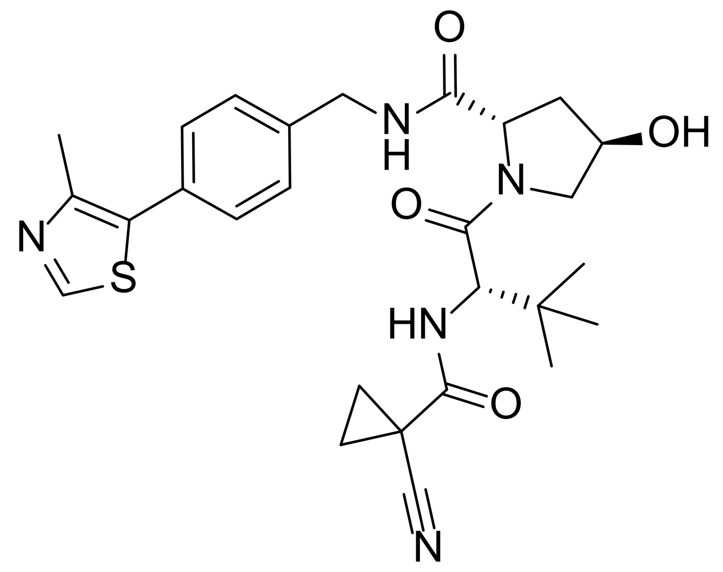	**523.23**
**VH032 (VHL ligand K_d 307 nM)**	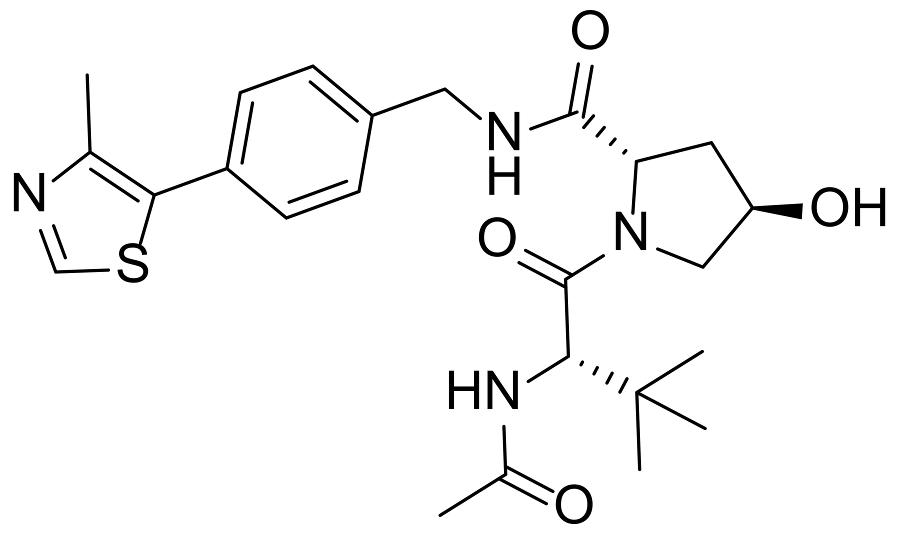	**472.21**

Table S3. Theoretical and experimental masses of all proteins and complexes detected by mass spectrometry

Complexes	Theoretical monoisotopic mass (Da)	Average experimental mass (Da)
**BCL-xL**	25,800	25,817[1]
**VCB Complex**	44,344	44,773
**EloC•EloB**	23,816	24,171
**EloC•VHL**	31,352	31,649
**EloB•VHL**	33,537	33,613
**BCL-xL•753b•VCB**	71,783	72,361
**BCL-xL•DT2216•VCB**	71,701	72,254
**BCL-xL•PZ32652•VCB**	71,783	72,516
**BCL-xL•753b**	27,456	27,491
**BCL-xL•DT2216**	27,357	27,384
**VCB•VH032**	27,456	45,303
**VCB•VH298**	44,916	45324
**BCL-xL•VCB**	70,144	70,786
**BCL-xL•753b•VHL•EloC**	58,791	59,204
**BCL-xL•753b•VHL•EloB**	60,976	61,457
**BCL-xL•753b•VHL**	47,984	48,223
**BCL-xL•VHL•EloB**	59,337	59,791
**BCL-xL•VHL•EloC**	57,152	57,551
**BCL-xL•VHL**	46,345	46,557
**BCL-xL•DT2216•VHL•EloC**	58,692	59,050
**BCL-xL•DT2216•VHL•EloB**	60,877	61,290
**BCL-xL•DT2216•VHL**	47,885	48,105

[1]The observed difference is intrinsic to the protein and not due to salt clustering or incomplete desolvation.

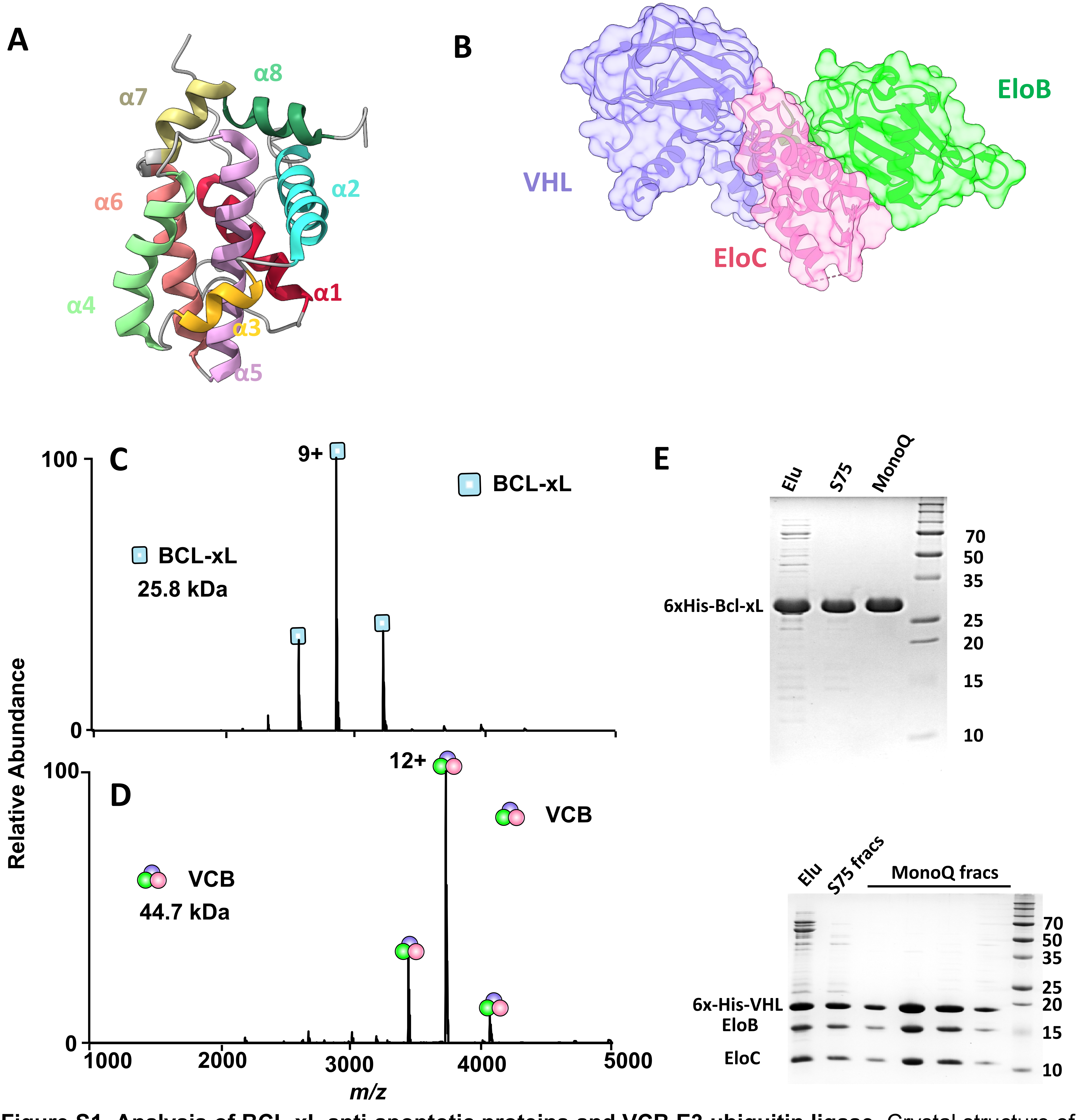

Figure S1. Analysis of BCL-xL anti-apoptotic proteins and VCB E3 ubiquitin ligase. Crystal structure of (A) BCL-xL (PDB: 1R2D) highlighting its helical regions and (B) the VCB complex (PDB: 8B0F) showing the three subunits: VHL (purple), EloB (green), and EloC (pink). MS1 spectra of solutions containing 10 μM of (C) BCL-xL, or (D) VCB E3 ligase after buffer exchange into 100 mM ammonium acetate, confirming the identity and purity of the expressed proteins. (E) SDS-PAGE gels showing the purification of (top) BCL-xL and (bottom) the VHL•EloC•EloB (VCB) complex.

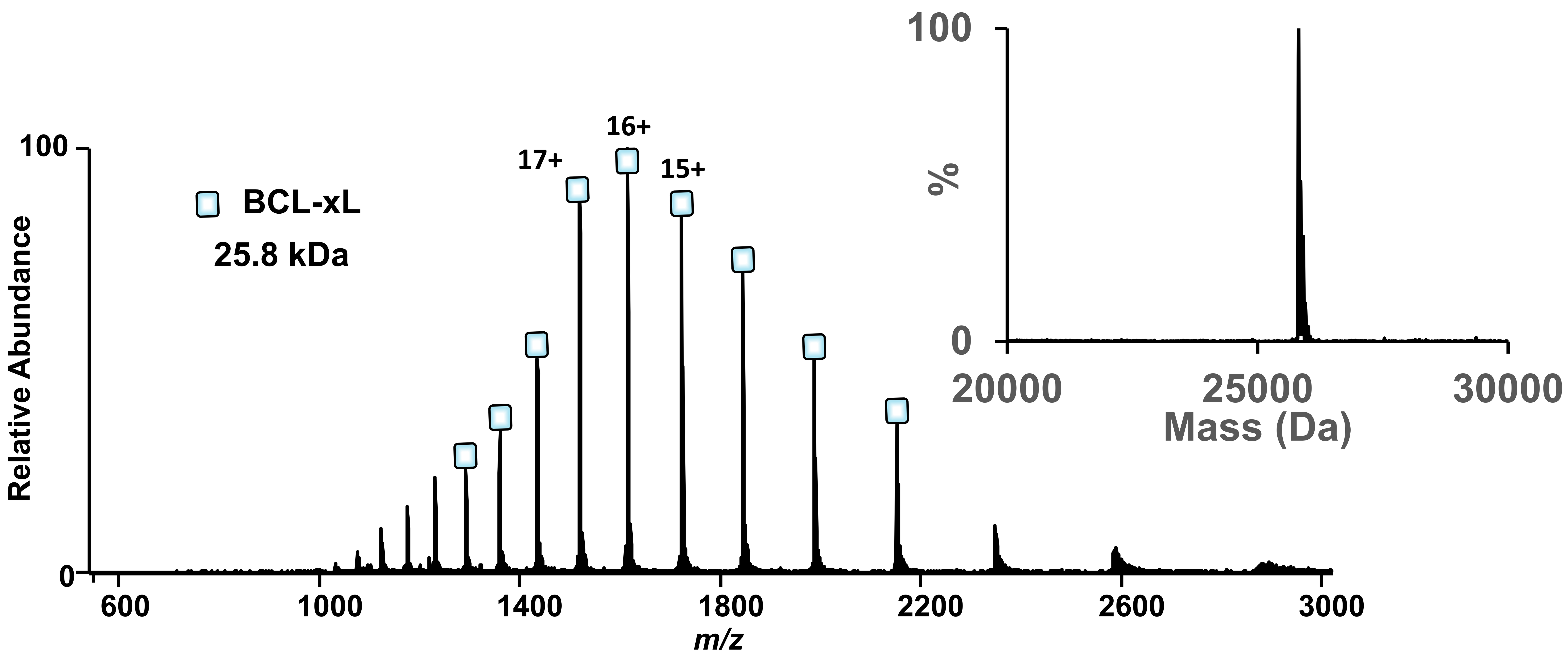

Figure S2. Analysis of BCL-xL proteins using denaturing conditions. MS1 spectra of solutions containing 5 µM of BCL-xL in 50% methanol containing 0.1% formic acid analyzed using an Orbitrap Fusion Lumos mass spectrometer confirming the identity and purity of the expressed proteins. The inset shows the corresponding deconvoluted mass spectrum.

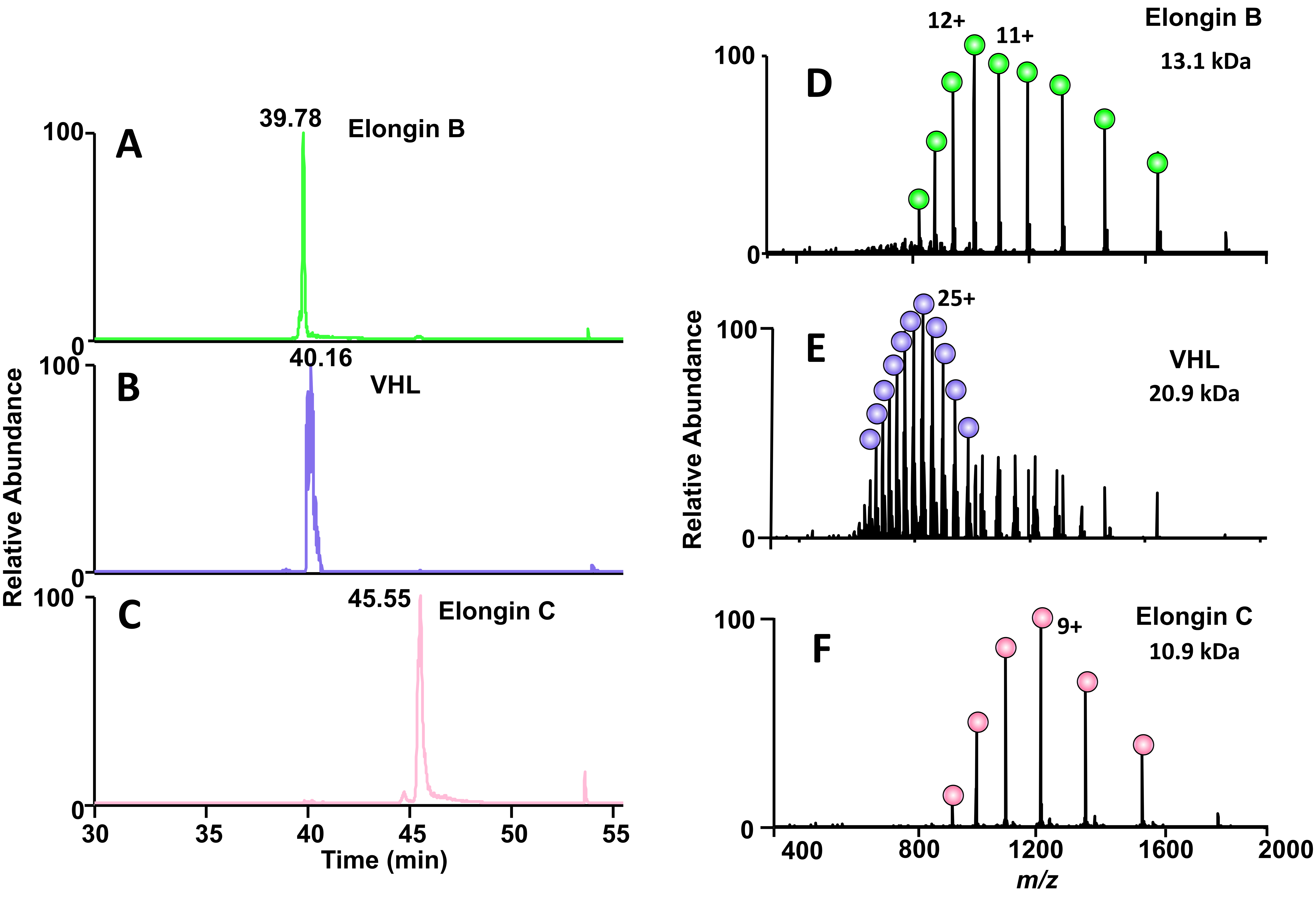

Figure S3. Characterization of the VCB E3 ligase protein complex using denaturing nano-LC-MS. Extracted ion chromatograms acquired from 30 to 55 minutes showing the separation of individual VCB subunits: (A) Elongin B (13.1 kDa), (B) VHL (20.9 kDa), and (C) Elongin C (10.9 kDa). (D-F) Corresponding mass spectra of the separated subunits acquired at the elution times shown on the left, confirming identity and purity.

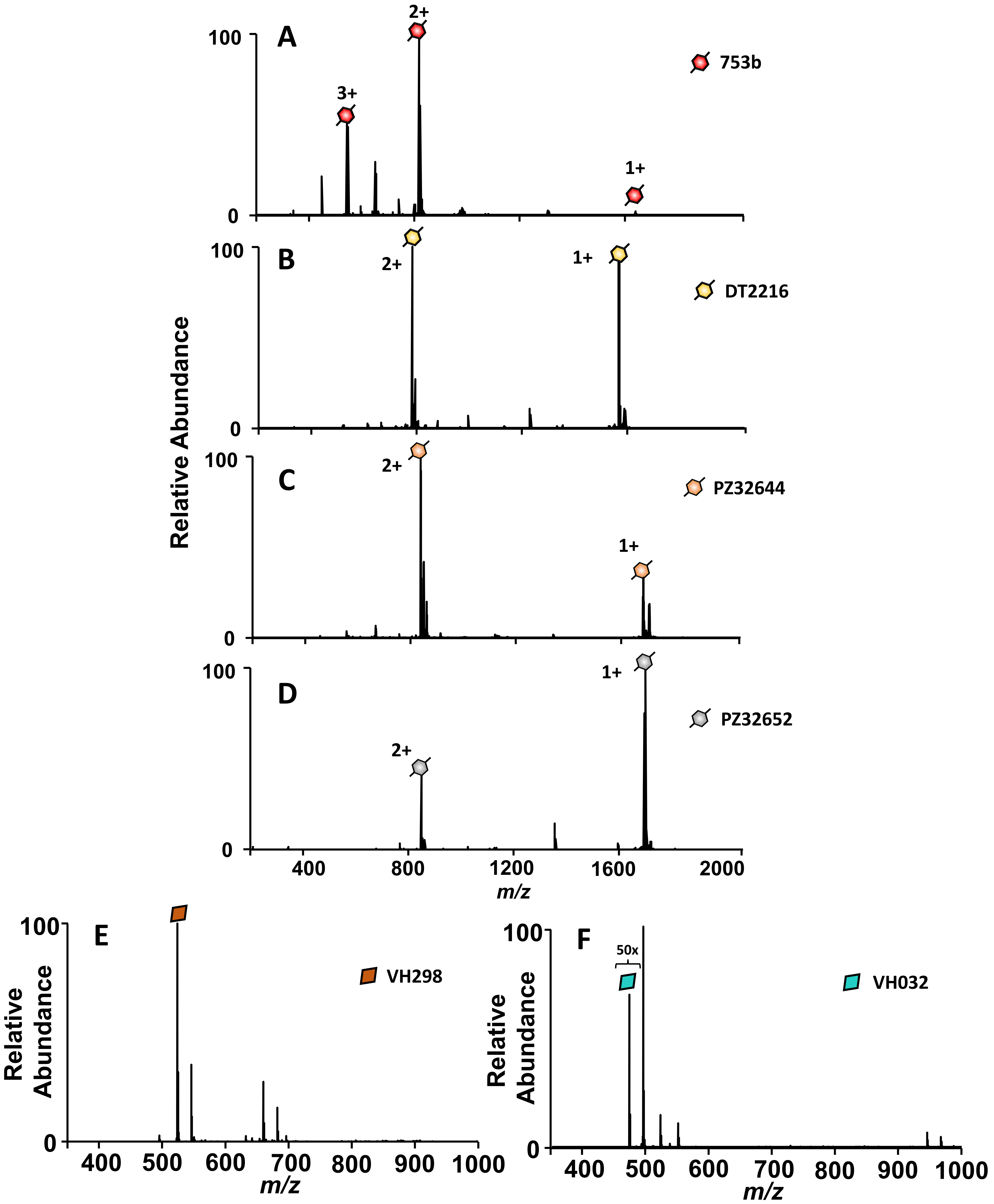

Figure S4. MS1 spectra of solutions containing 5 µM of a PROTAC or VHL inhibitor acquired on a Thermo HFX Orbitrap mass spectrometer. Panels A–D show spectra for the four PROTACs: (A) 753b (B) DT2216 (C) PZ32644 and (D) PZ32652. Panels E–F show spectra for the VHL inhibitors: (E) VH298 and (F) VH032 (the latter mainly as a sodium adduct). All spectra were collected in methanol with 0.1% formic acid using a spray voltage of 0.8 kV and a resolution setting of 45,000.

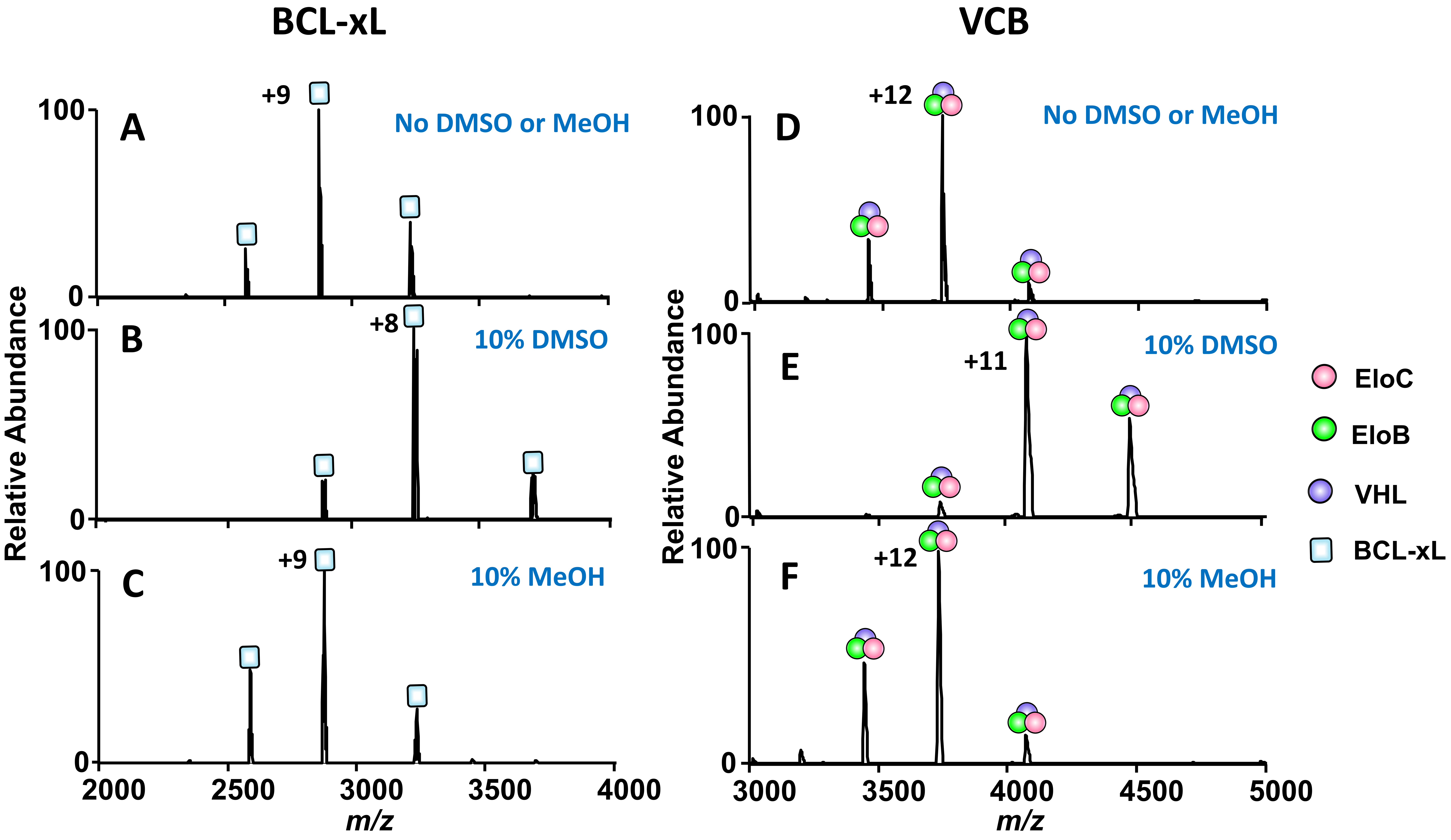

Figure S5. MS1 spectra of BCL-xL and VCB E3 ligase in different solvent conditions. MS1 spectra of 10 μM of BCL-xL (left-side) or VCB E3 ligase (right-side) sprayed from 100 mM ammonium acetate (A and D) without or with the addition of (B and E) 10% DMSO or (C and F) 10% MeOH. The addition of 10% DMSO results in a slight shift towards lower charge states.[1] In contrast, the addition of 10% MeOH does not significantly alter the charge state distribution.

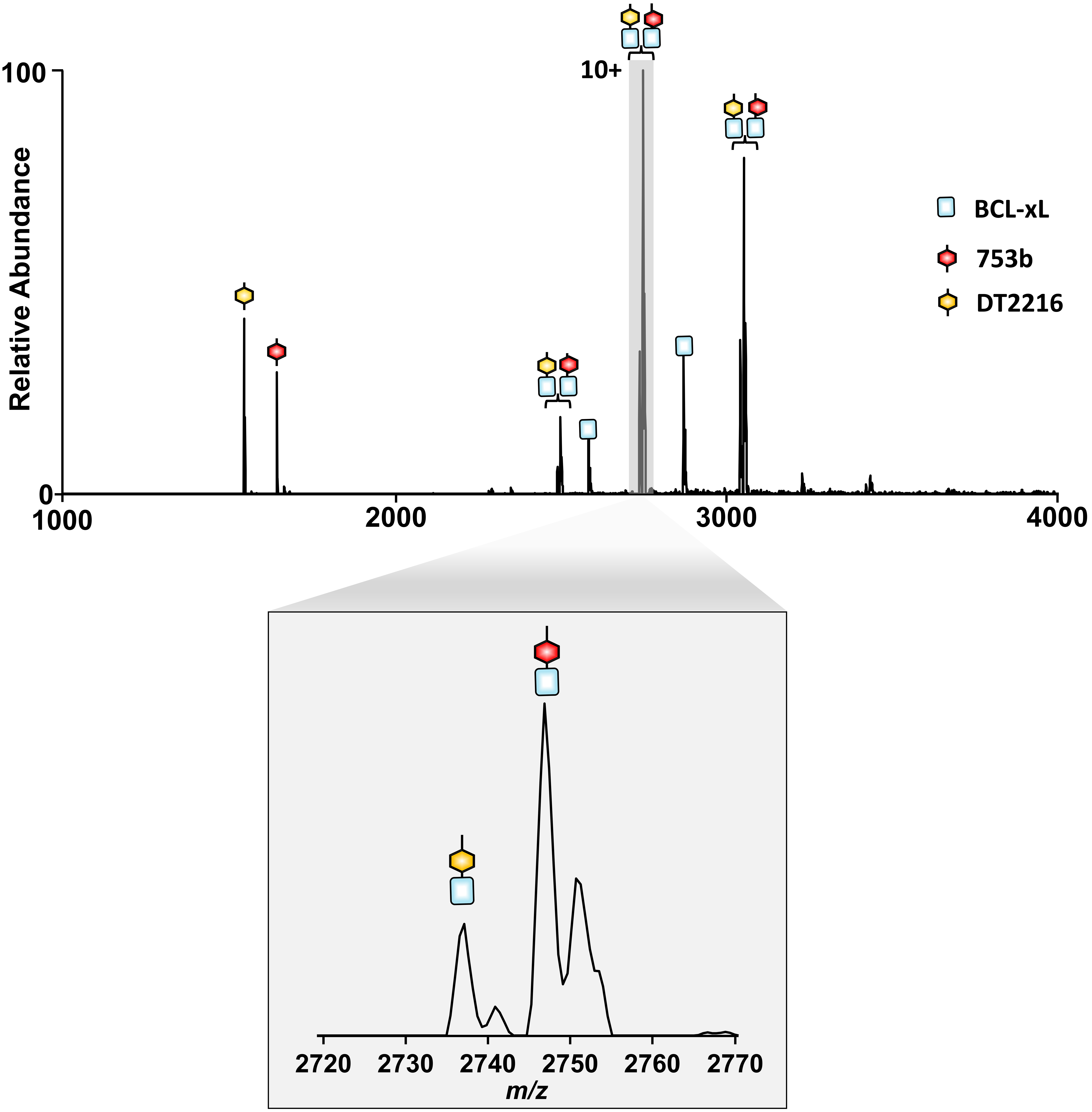

Figure S6. Direct competition experiment demonstrates preferential binding of 753b over DT2216 to BCL-xL. Native MS spectrum of BCL-xL incubated simultaneously with equimolar 753b and DT2216 (10 µM each). The spectrum shows dominant formation of the BCL-xL•753b binary complexes with lower formation of the BCL-xL•DT2216 complexes, supporting that 753b outcompetes DT2216 for BCL-xL binding under identical conditions.

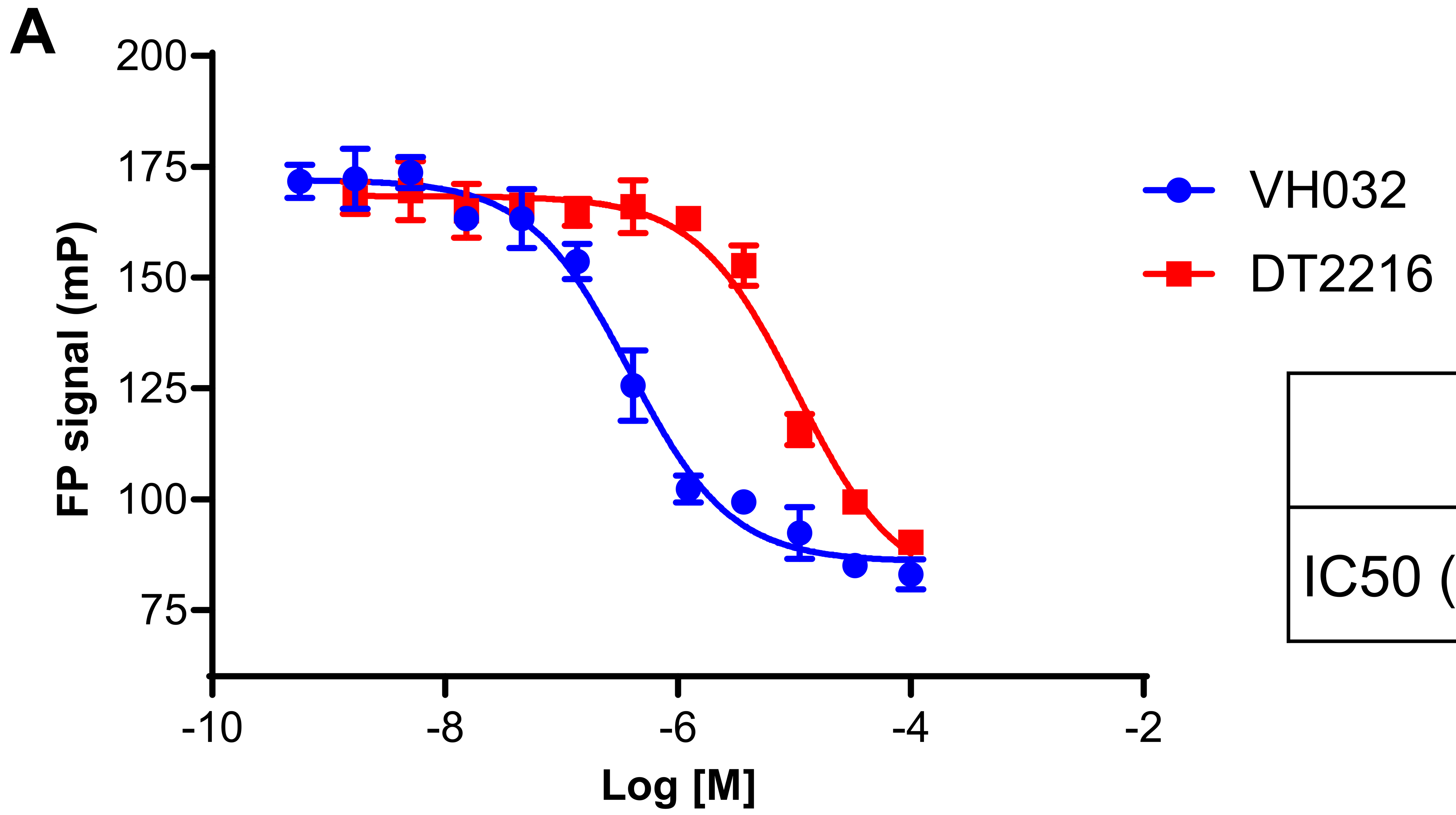

	VH032	DT2216
IC50 (μM)	0.386	10.6

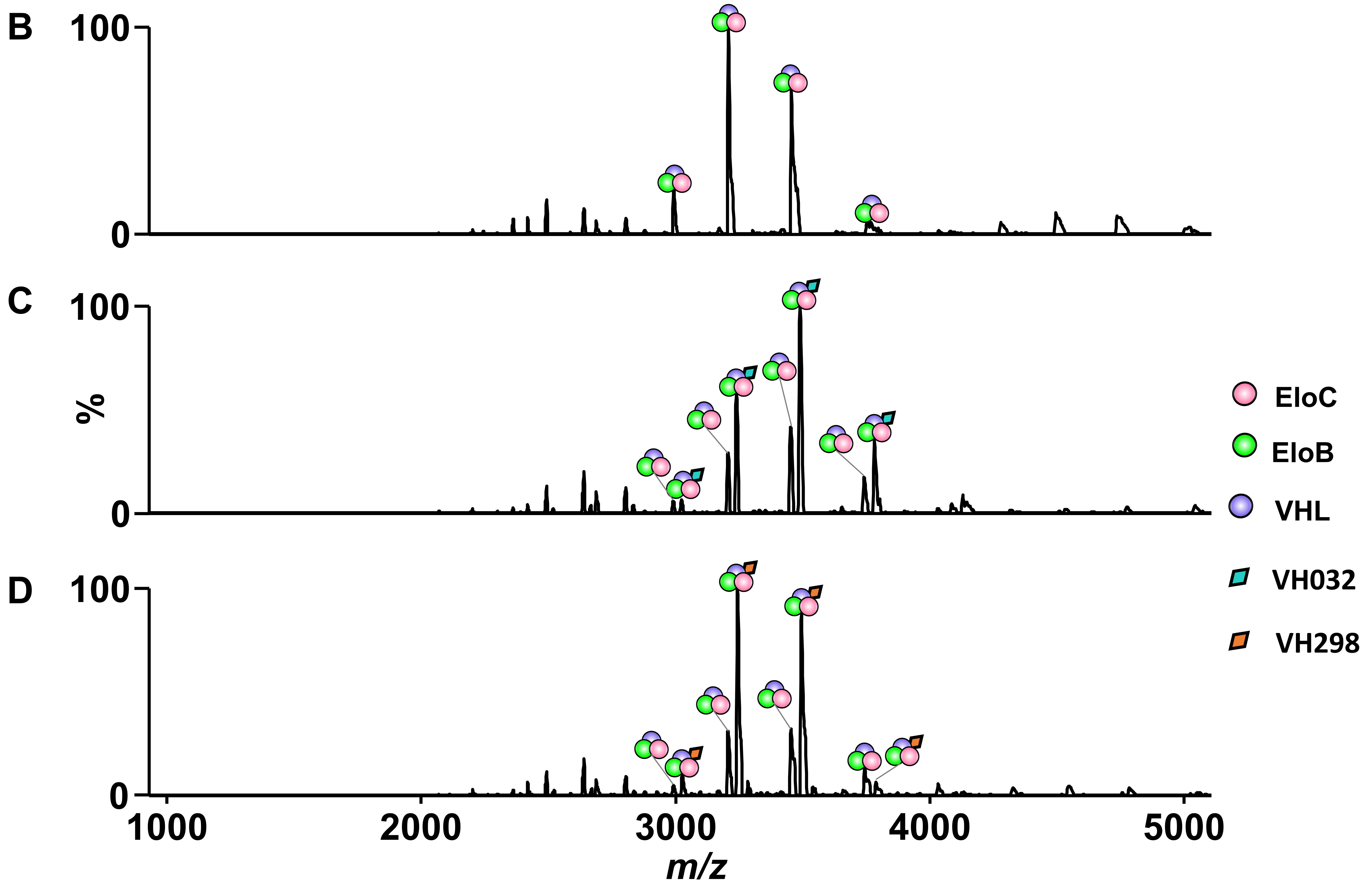

Figure S7. Evaluation of VCB binding to PROTAC DT2216 and high-affinity VHL Inhibitors. (A) Fluorescence polarization (FP) binding curves for DT2216 and VH032 measured against VCB. The low DT2216–VCB affinity explains the absence of detectable binary PROTAC–VCB complexes in the native ESI mass spectra. Native mass spectra acquired under identical conditions as used for the other PROTAC experiments for solutions containing: B) 10 μM VCB alone, C) 10 μM VCB and 10 μM VH032 (IC50 = 185 nM) and D) 10 μM VCB and 10 μM VH298 (IC50 = 90 nM).[2] Both high-affinity inhibitors produce abundant binary VCB•ligand complexes, demonstrating that the method readily detects interactions with VCB when the ligand affinity is sufficiently high, unlike the lack of complexes observed between VCB and 753b, DT2216, or PZ32652, in **Figure 1B**.

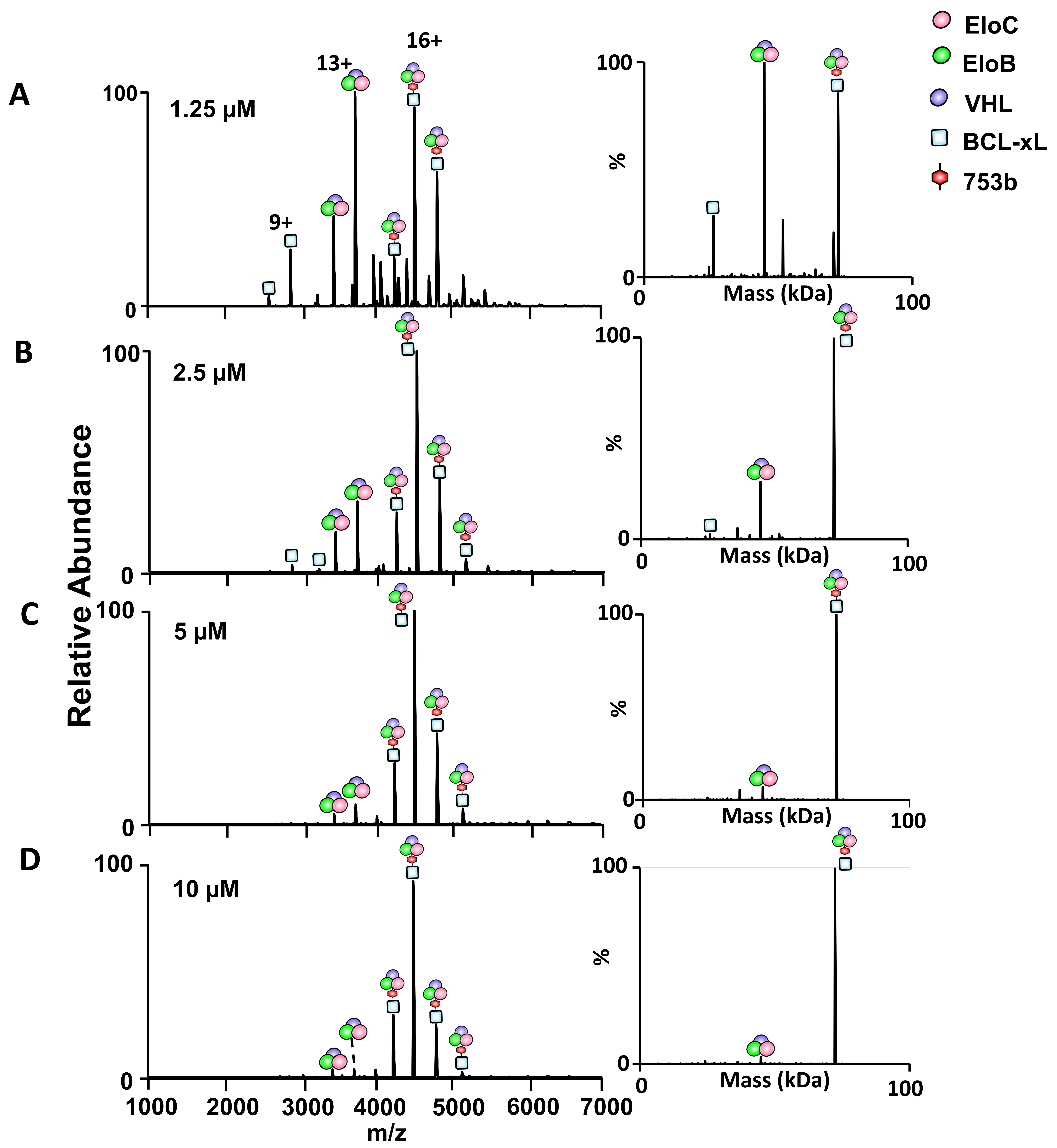

Figure S8. Titration of BCL-xL and VCB with 753b PROTAC monitored using native MS. (A) ESI mass spectra obtained for solutions containing 5 µM BCL-xL and 5 µM VCB in 100 mM ammonium acetate with various concentrations of 753b: A) 1.25 µM, B) 2.5 µM, C) 5 µM, or D) 10 µM. The corresponding deconvoluted spectra are shown on the right. The spectra show an increase in the relative abundances of ternary BCL-xL•753b•VCB complexes as the PROTAC concentration increases.

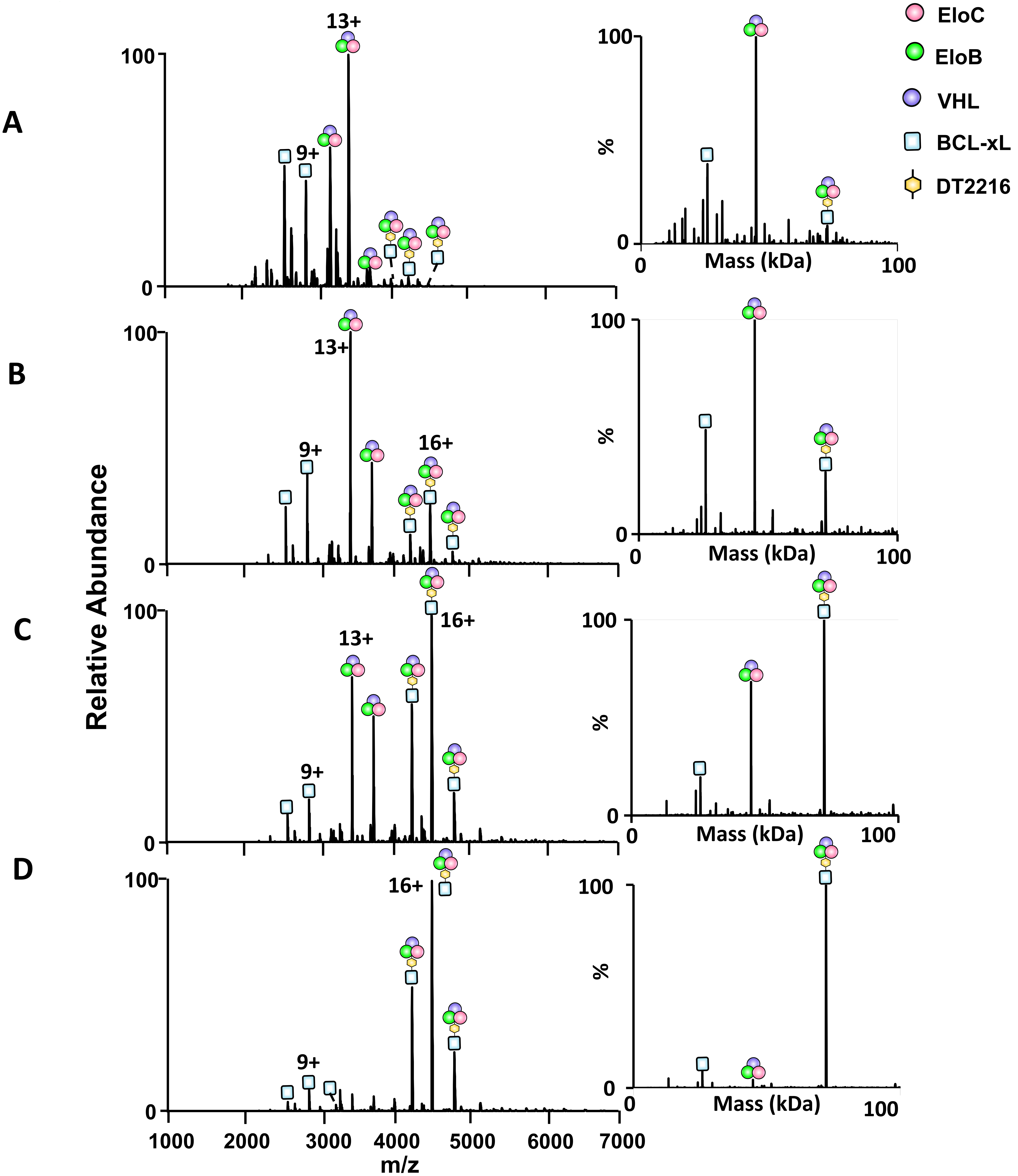

Figure S9. Titration of BCL-xL and VCB with DT2216 PROTAC monitored using native MS. (A) ESI mass spectra obtained for solutions containing 5 µM BCL-xL and 5 µM VCB in 100 mM ammonium acetate with various concentrations of DT2216: A) 1.25 µM, B) 2.5 µM, C) 5 µM, or D) 10 µM. The corresponding deconvoluted spectra are shown on the right. The spectra show an increase in the relative abundances of ternary BCL-xL•DT2216•VCB complexes as the PROTAC concentration increases.

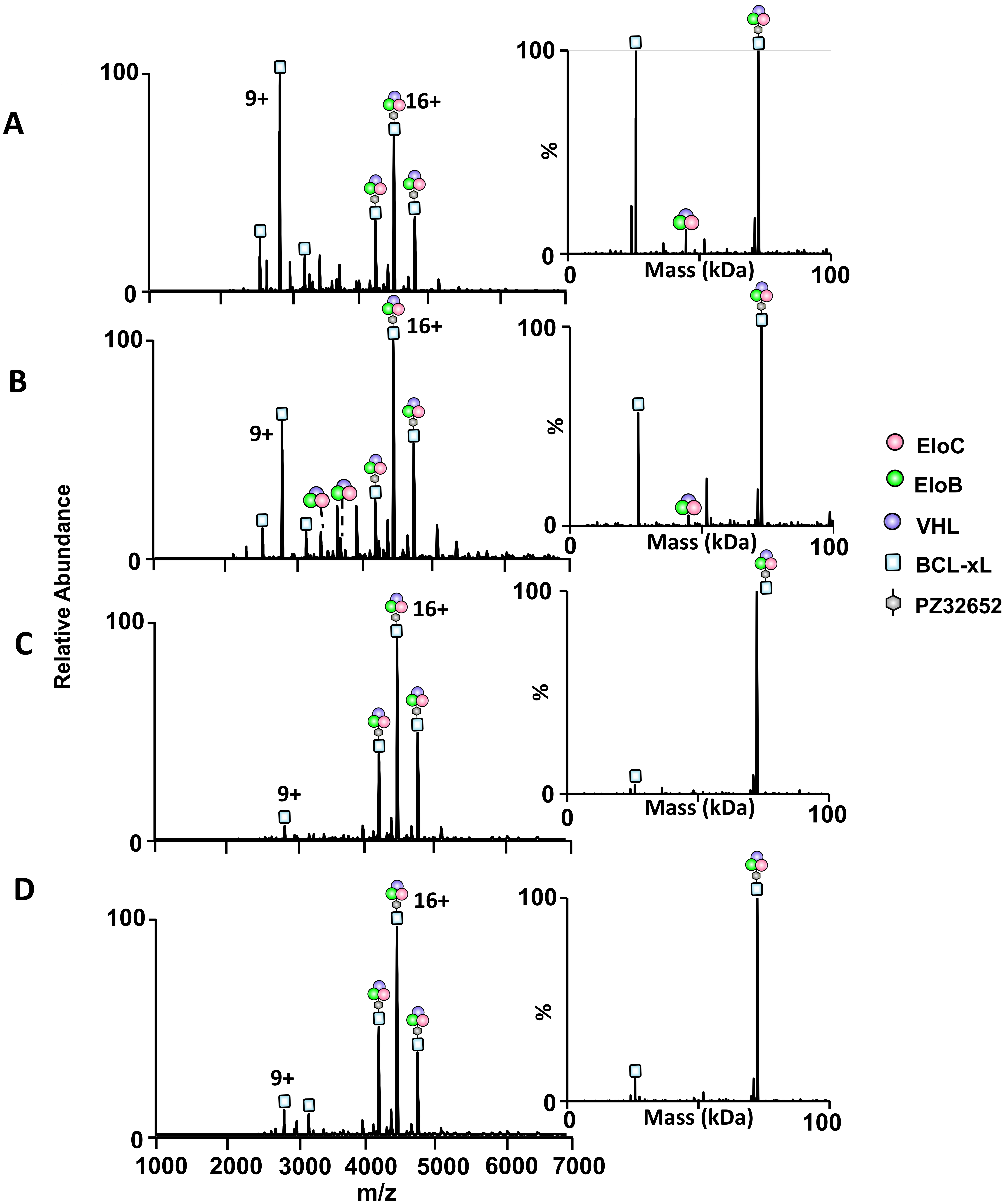

Figure S10. Titration of BCL-xL and VCB with PZ32652 PROTAC using native MS. (A) ESI mass spectra obtained for solutions containing 5 µM BCL-xL and 5 µM VCB in 100 mM ammonium acetate and various concentrations of PZ32652: A) 1.25 µM, B) 2.5 µM, C) 5 µM, or D) 10 µM. The corresponding deconvoluted spectra are shown on the right. The spectra show an increase in the relative abundances of ternary BCL-xL• PZ32652 •VCB complexes as the PROTAC concentration increases.

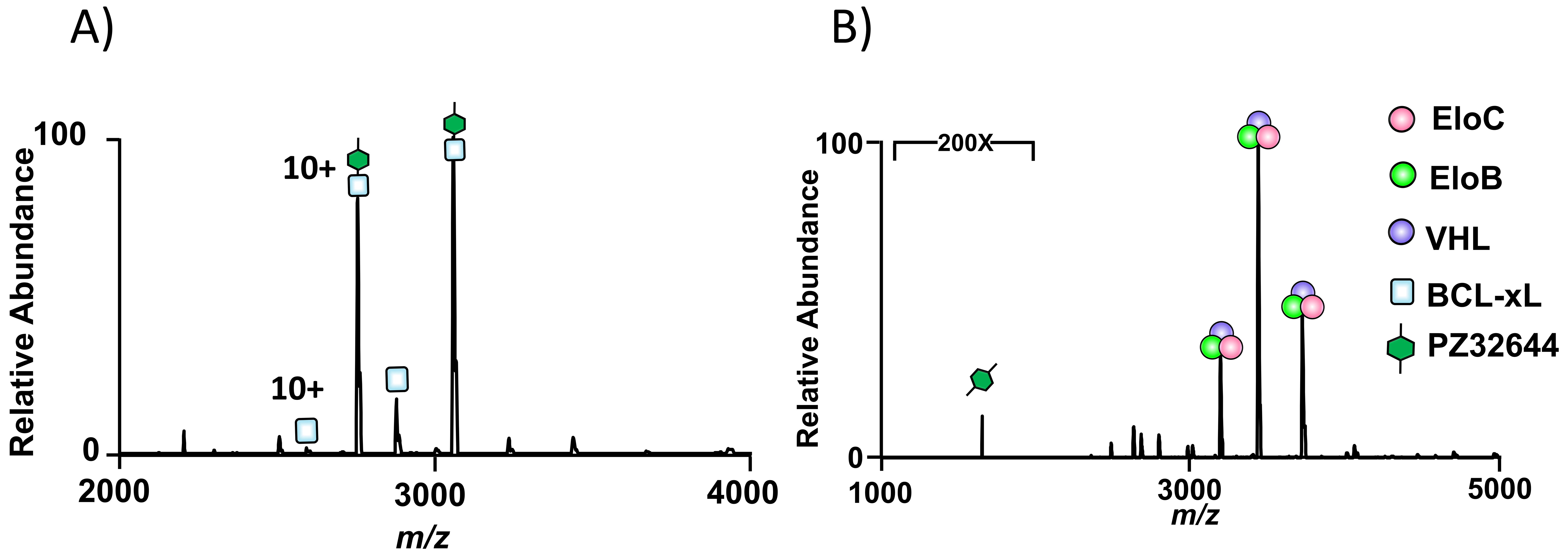

Figure S11. Binary interactions of negative control PROTAC PZ32644 with BCL-xL and VCB evaluated by native MS. ESI mass spectra obtained for solutions containing 10 µM of (A) BCL-xL or (B) VCB E3 ligase complex after incubation with 10 µM PROTAC PZ32644. This PROTAC showed binding to BCL-xL, but no detectable interactions were observed with the VCB ligase complex for PZ32644.

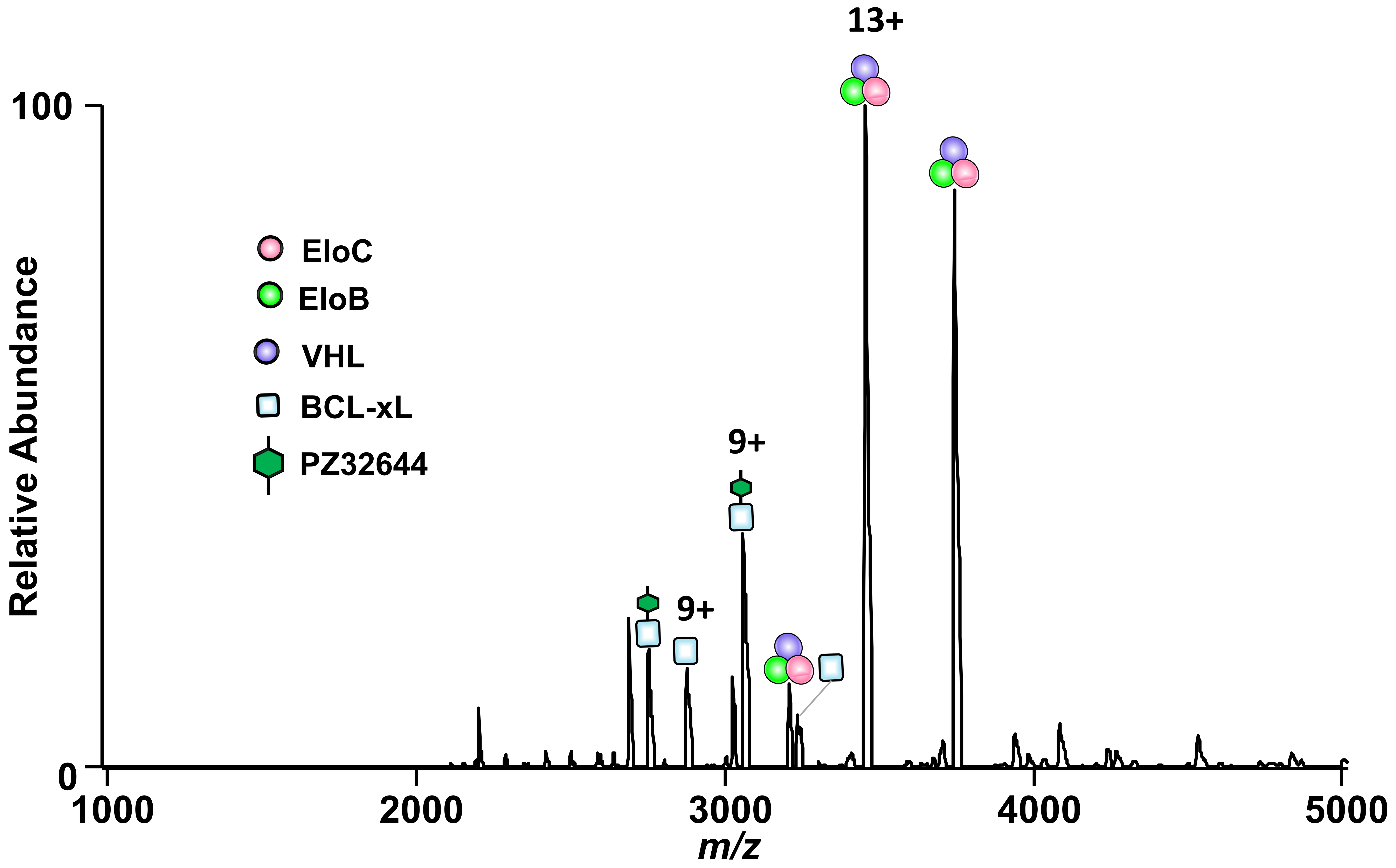

Figure S12. PROTAC PZ32644 does not induce ternary complex formation. (A) MS1 spectrum of a solution containing 5 µM BCL-xL and 5 µM VCB E3 ligase incubated with 5 µM of a non-binding PROTAC (PZ32644), following buffer exchange into 100 mM ammonium acetate. No ions corresponding to ternary complexes were observed, confirming the inability of this PROTAC to promote complex formation. However, this PROTAC does form complexes with BCL-xL as also seen in **Figure S9A**.

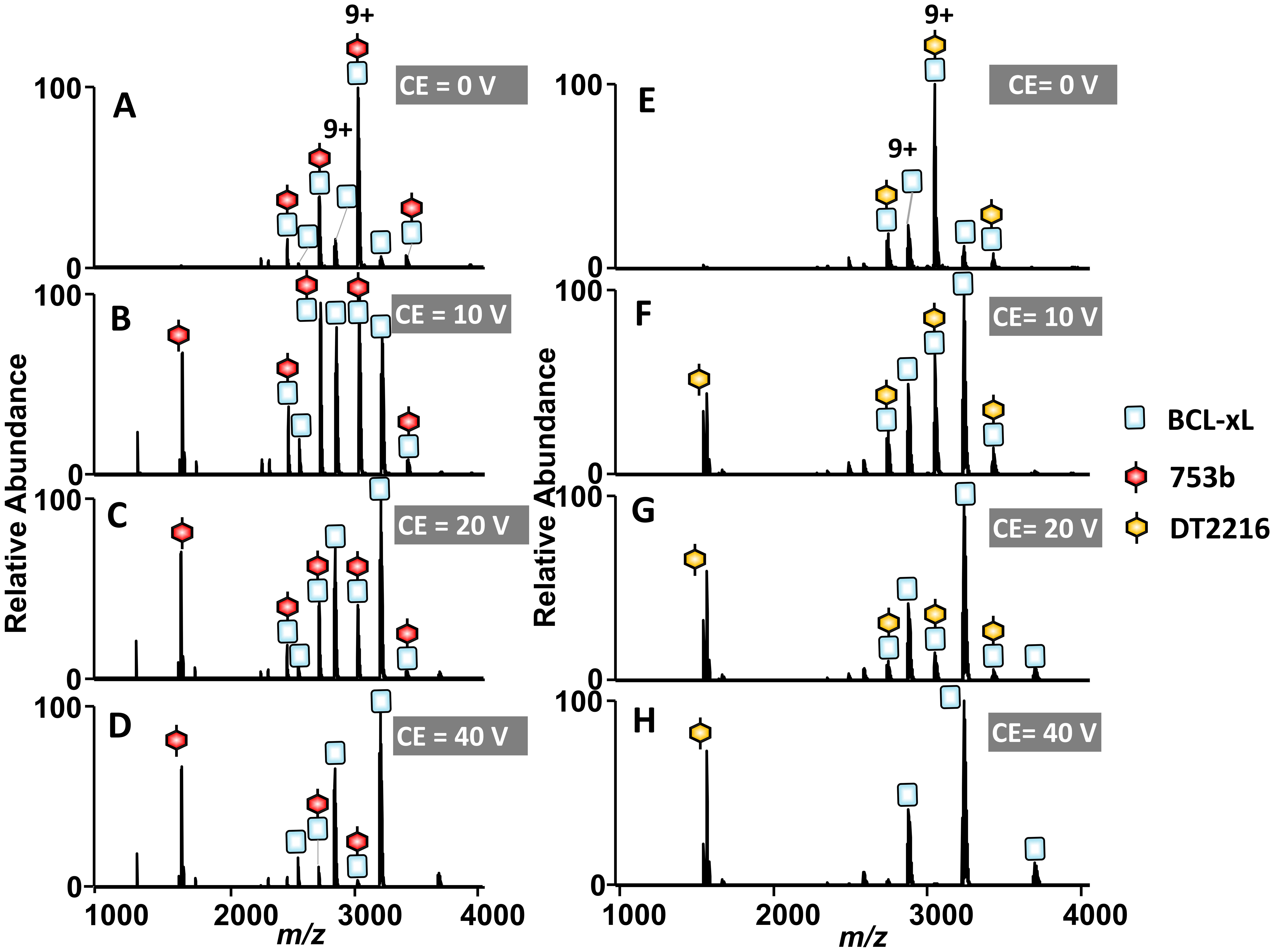

Figure S13. Evaluation of the gas-phase stabilities of binary BCL-xL•PROTAC complexes based on energy-variable CID. CID mass spectra of BCL-xL•PROTAC complexes (co-isolated 8+ to 11+ charge states) containing (A-D) 753b or (E-H) DT2216 acquired at selected collision voltages. Spectra were collected at additional intermediate voltages but are omitted here for clarity. The 753b complex shows greater resistance to dissociation, indicating higher kinetic stability and stronger interaction with BCL-xL in the gas phase compared to DT2216.

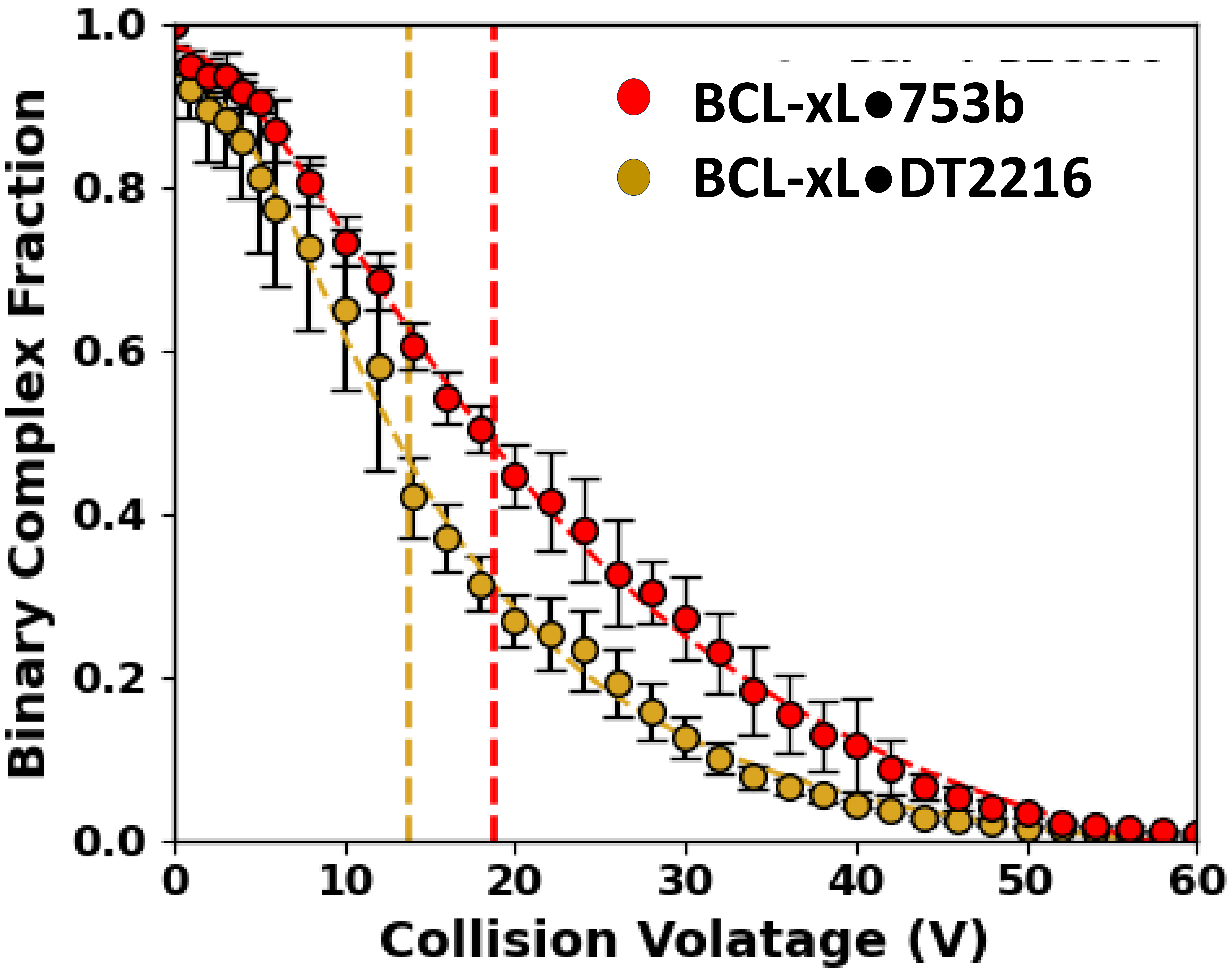

Figure S14. Energy-variable CID curves for the co-isolated +8 to +10 charge states of the binary complexes BCL-xL•753b (red) and BCL-xL•DT2216 (gold) with the CE_{50} values marked with dashed lines. The MS/MS parameters were identical, and the experiments were run back-to-back for the solutions containing each PROTAC.

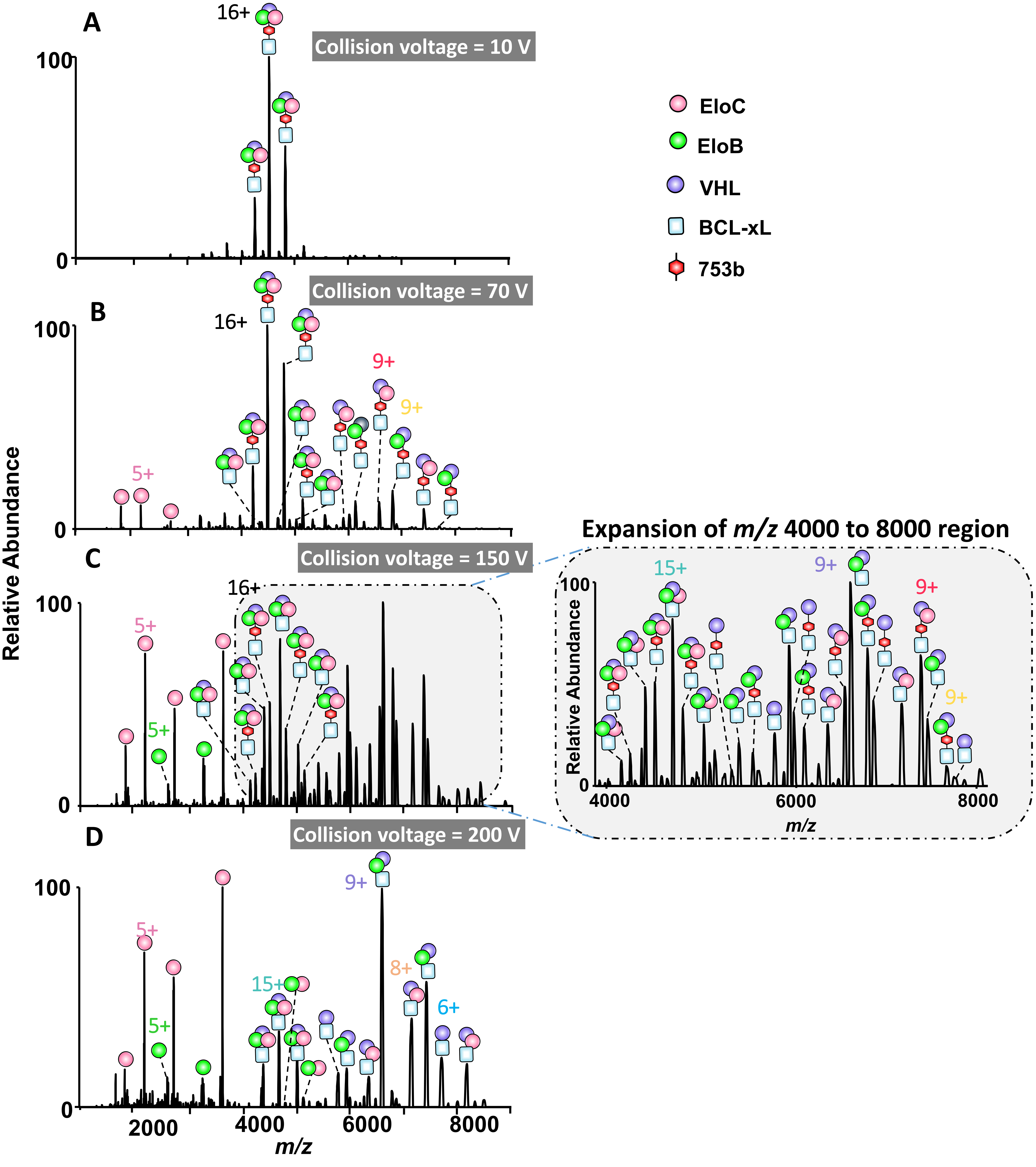

Figure S15. Energy-variable CID of ternary BCL-xL•753b•VCB complexes. CID mass spectra of the BCL-xL•753b•VCB ternary complex (co-isolated 14+ to 17+ charge states) using CID voltages of: (A) 10 V, (B) 70 V, (C) 150 V, and (D) 200 V.

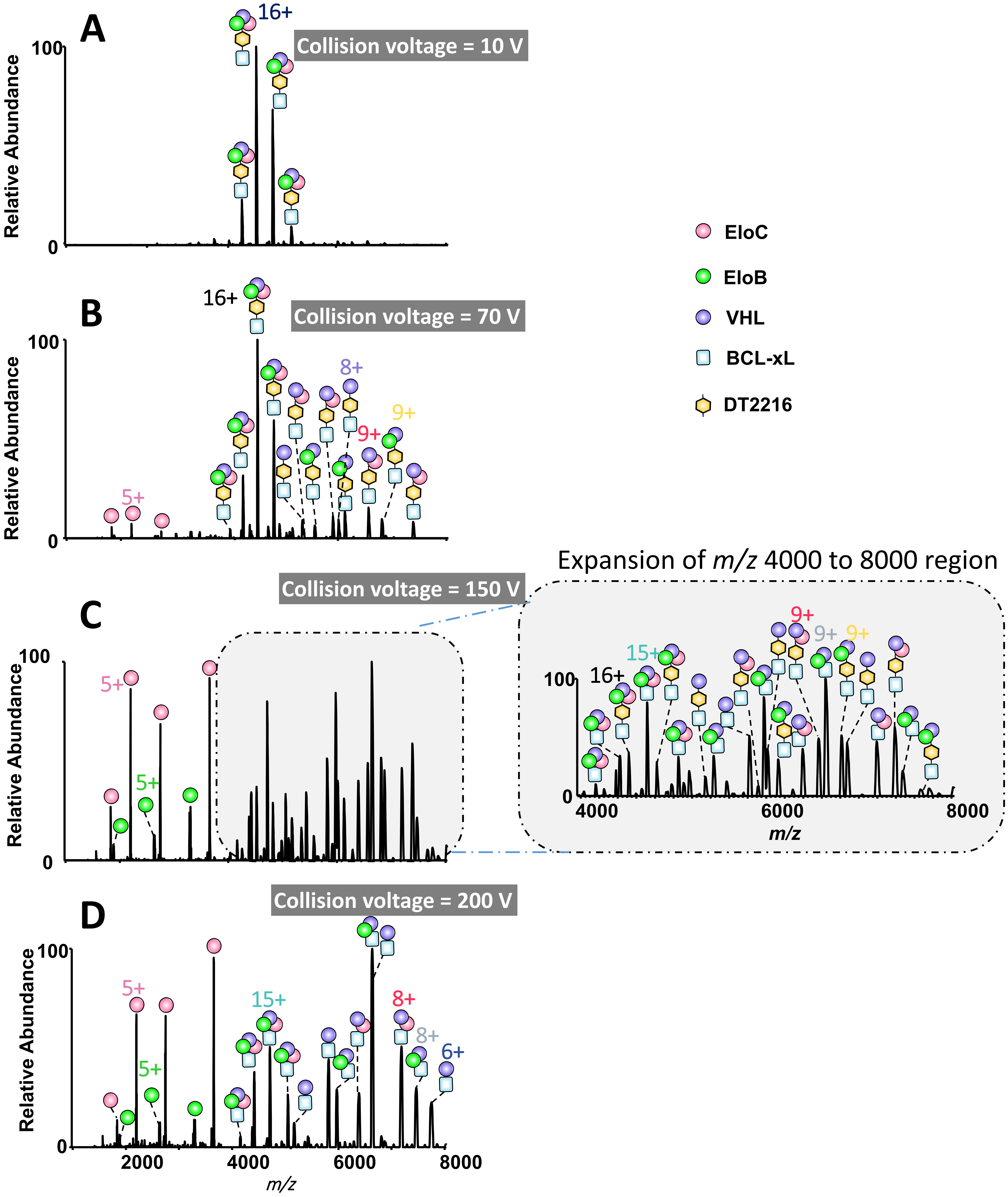

Figure S16. Energy-variable CID of ternary BCL-xL•DT2216•VCB complexes. CID mass spectra of the BCL-xL•DT2216•VCB ternary complex (co-isolated 14+ to 17+ charge states) using CID voltages of: (A) 10 V, (B) 70 V, (C) 150 V, and (D) 200 V.

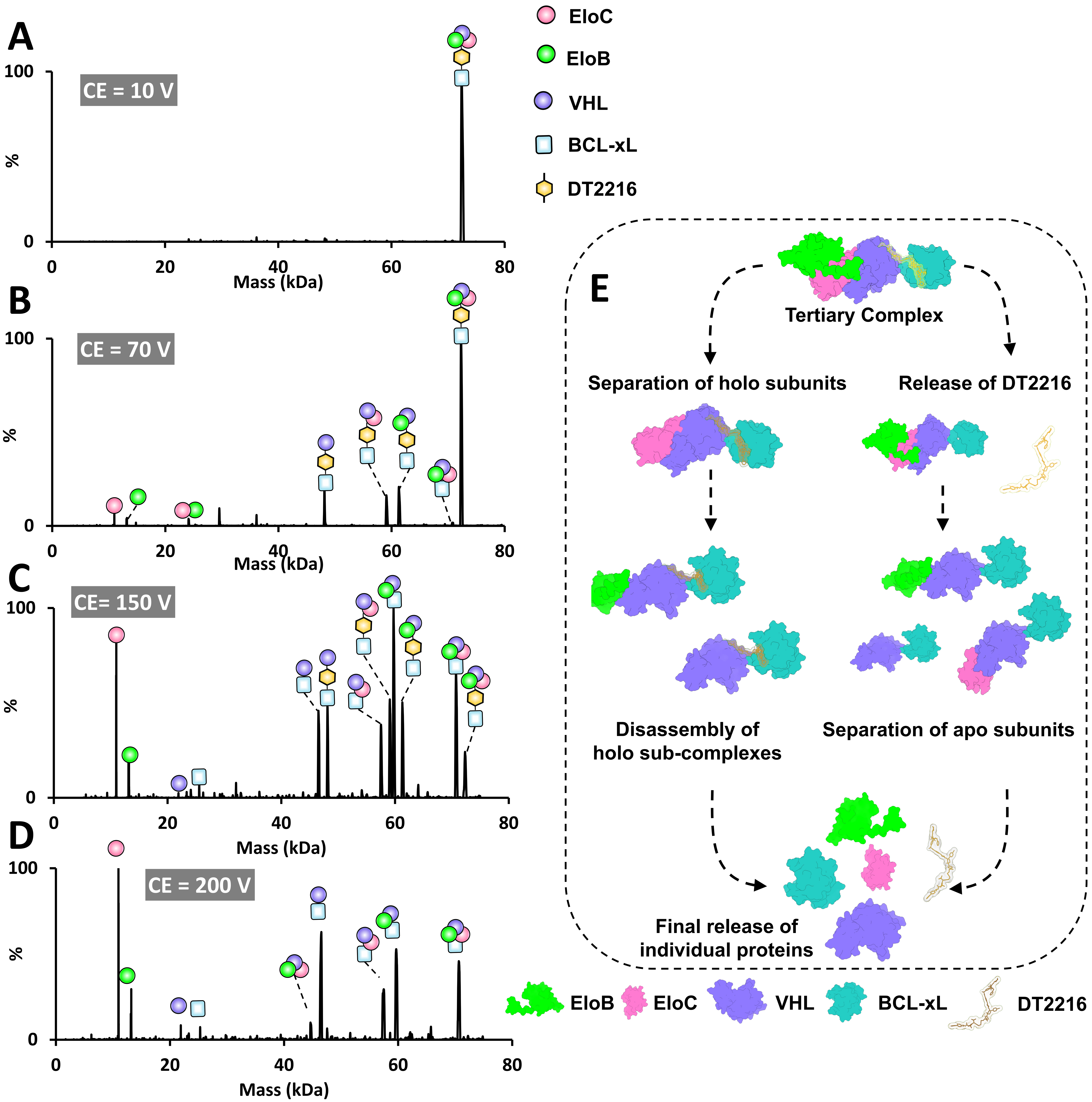

Figure S17. Collision-induced dissociation of ternary BCL-xL•DT2216•VCB complexes reveals two distinct dissociation pathways. (A-D) Deconvoluted CID mass spectra obtained from ESI-MS/MS analysis of solutions containing 5 μM BCL-xL and 5 μM VCB incubated with 5 μM of DT2216 (co-isolated 14+ to 17+ charge states) as a function of CID collision energy. The ternary complexes dissociate in a stepwise manner by two pathways as the collision energy increases. (E) Schematic representation of the two dissociation pathways observed during CID. The holo-subcomplexes retains PROTAC-mediated interactions between VHL and BCL-xL (left-side), whereas the apo-subcomplex results from the direct release of DT2216 (right-side).

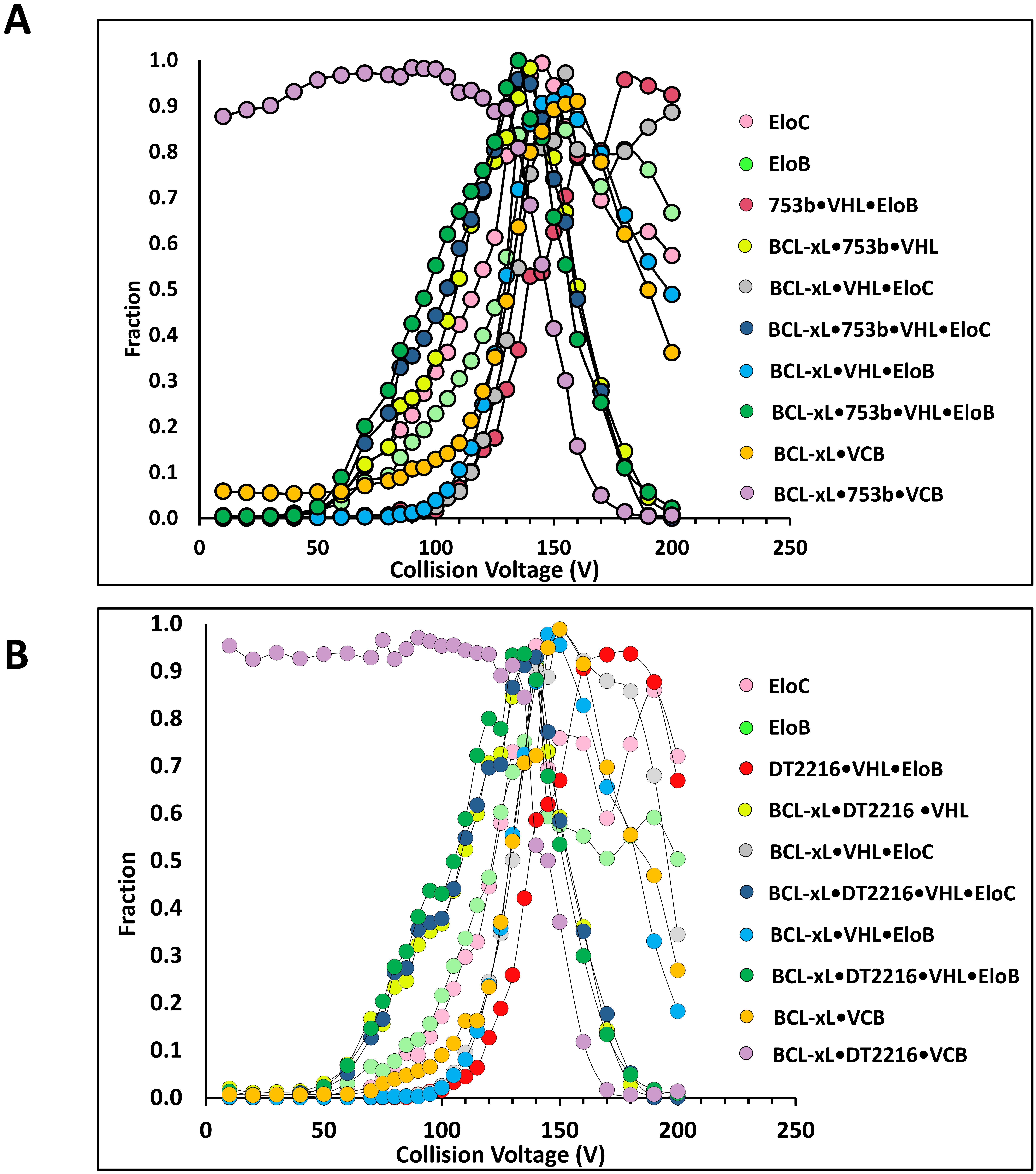

Figure S18. Product ion distributions based on energy-variable CID of PROTAC-mediated ternary complexes for (A) BCL-xL•753b•VCB (co-isolated 14+ to 17+ charge states) and (B) BCL-xL•DT2216•VCB (co-isolated 14+ to 17+ charge states). The resulting subcomplexes and monomeric subunits were tracked as a function of increasing collision energy. The curves were constructed using UniDec deconvolution of each CID spectrum, followed by extraction of the integrated peak areas corresponding to the intact ternary complex and all resolved subcomplexes. For each collision voltage, the ion peak areas of these assigned species were summed, and the abundances of each species were normalized to this total signal. Only native-like subcomplexes identified by UniDec were included. Fragment ions (which are not mass-resolved into native-state species) were not included in the quantitative analysis. Dissociation profiles of the individual ions are shown in **Figure S19**.

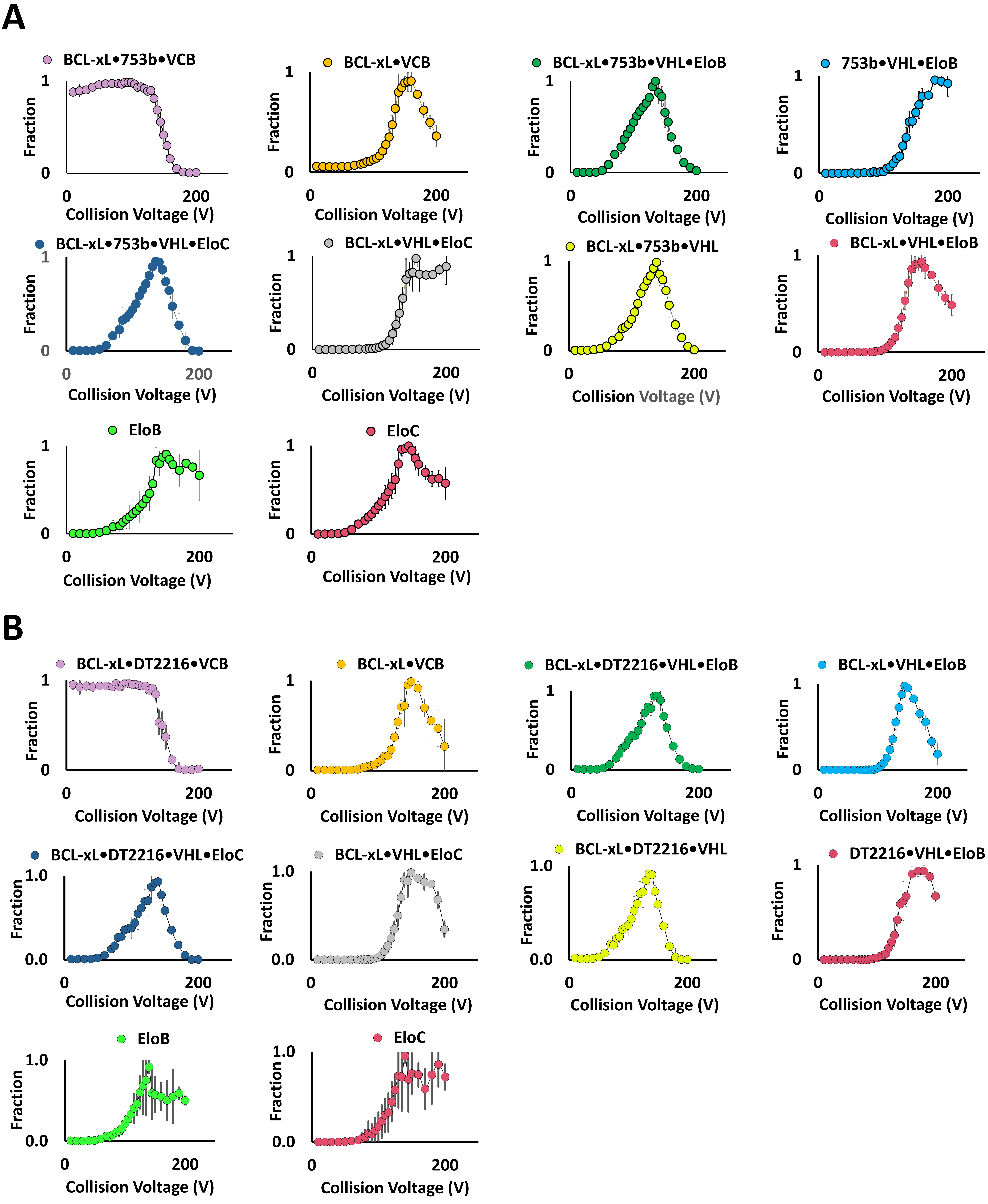

Figure S19. Dissociation profiles of the individual ions tracked in **Figure S18**. (A) BCL-xL•753b•VCB (co-isolated 14+ to 17+ charge states) and (B) BCL-xL•DT2216•VCB (co-isolated 14+ to 17+ charge states).

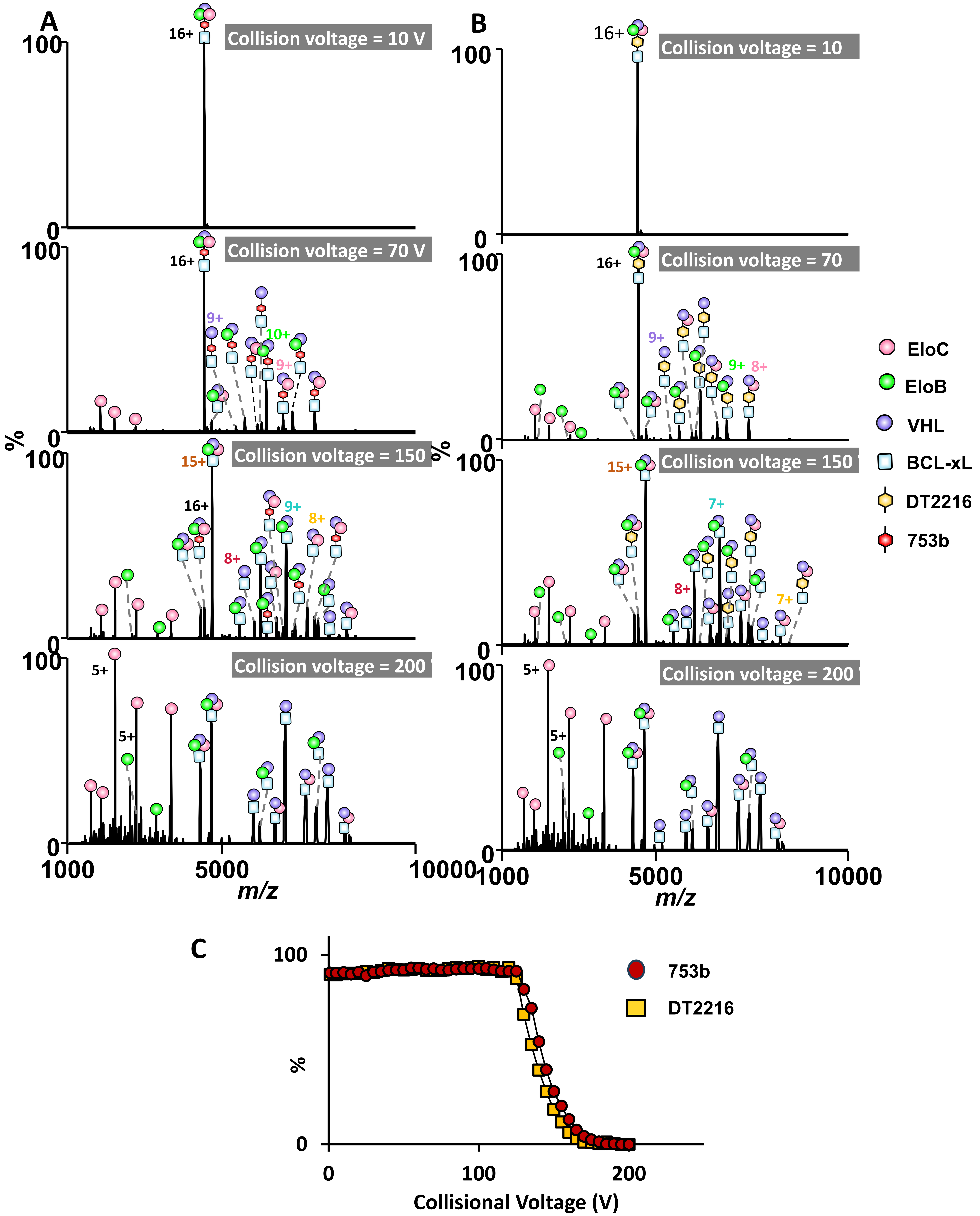

Figure S20. Energy-variable CID of the 16+ charge state of (A) BCL-xL·753b·VCB and (B) BCL-xL·DT2216·VCB ternary complexes generated from solutions containing 5 μM BCL-xL and 5 μM VCB incubated with 5 μM of the respective PROTAC. (C) Energy-variable CID breakdown graph displaying the abundance of the precursor as the collision energy is varied. Both ternary complexes exhibited comparable dissociation behavior and kinetic stabilities.

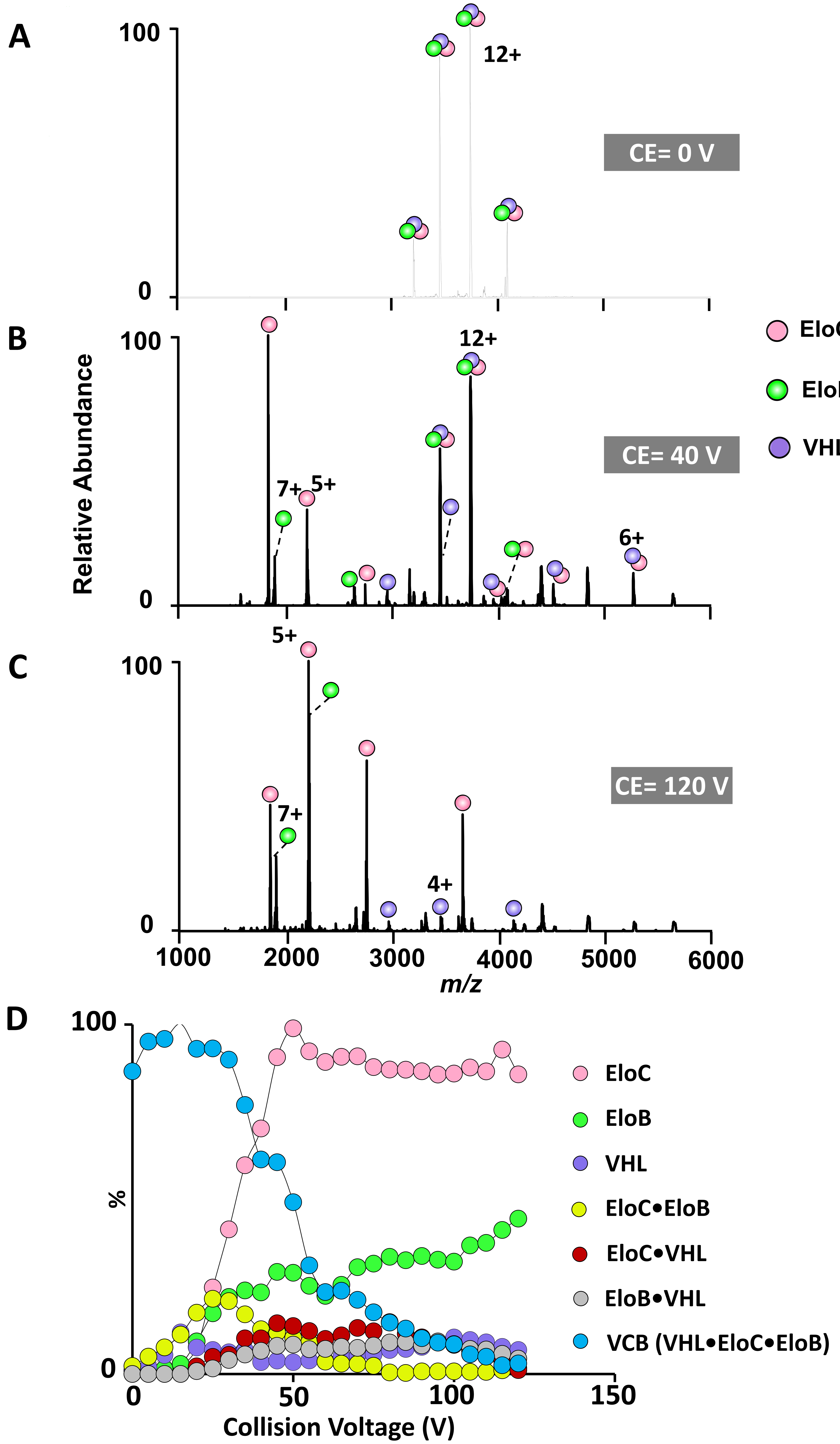

Figure S21. Dissociation behavior of the VCB E3 ligase complexes as a function of CID collision voltage. CID mass spectra of the VCB complexes (co-isolated 11+ to 14+ charge states) acquired using different CID voltages, revealing the progressive release of subunits EloB and EloC. (D) The relative abundances of individual subunits and subcomplexes are plotted as a function of CID collision voltage, demonstrating stepwise dissociation. EloC is released first, followed by EloB and VHL, reflecting the binding hierarchy in the VCB assembly. Dissociation profiles of the individual ions are shown in **Figure S22.**

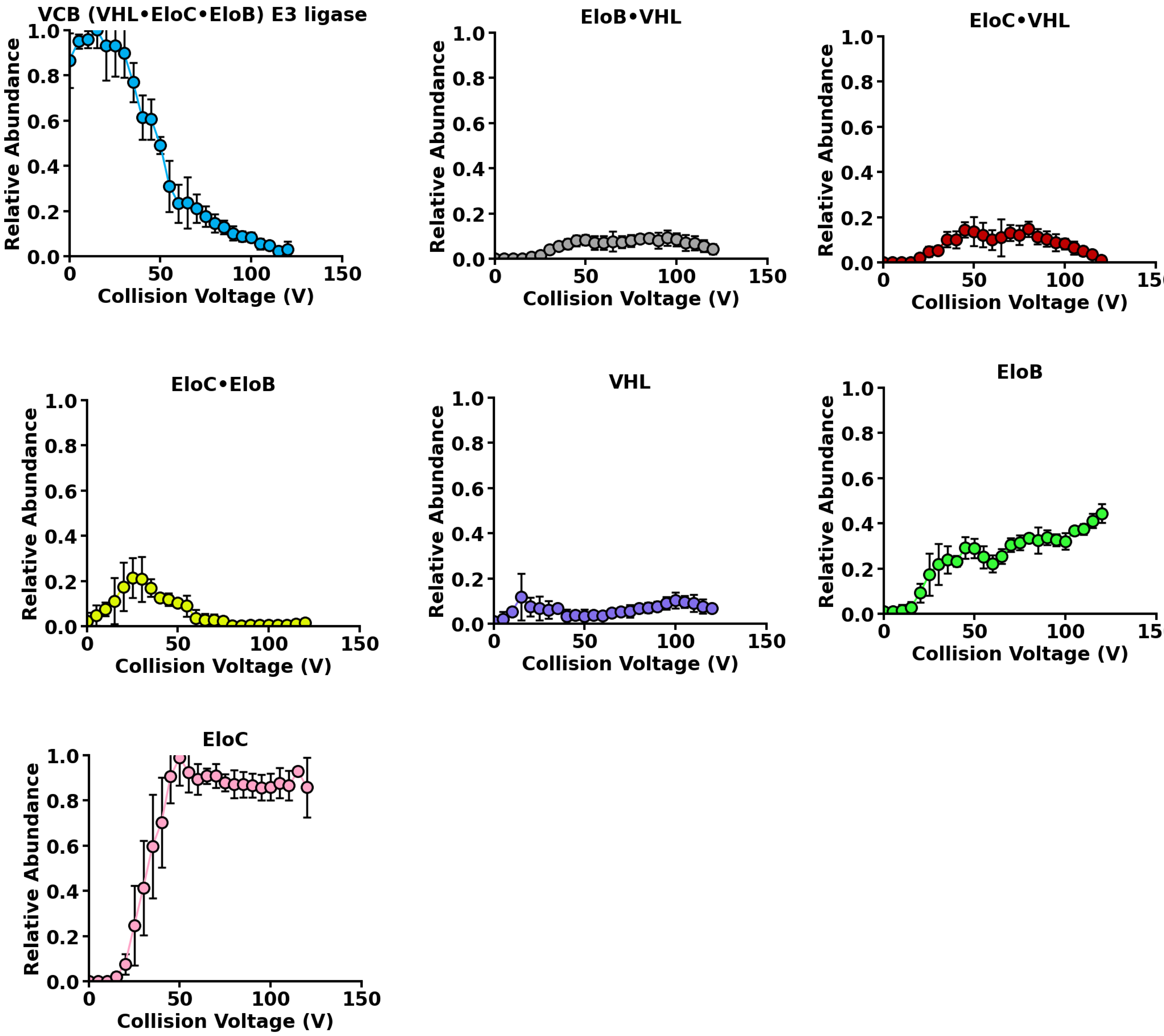

Figure S22. Dissociation profiles of the individual ions tracked in **Figure S21 for** VCB (VHL•EloC•EloB) (co-isolated 11+ to 14+ charge states).

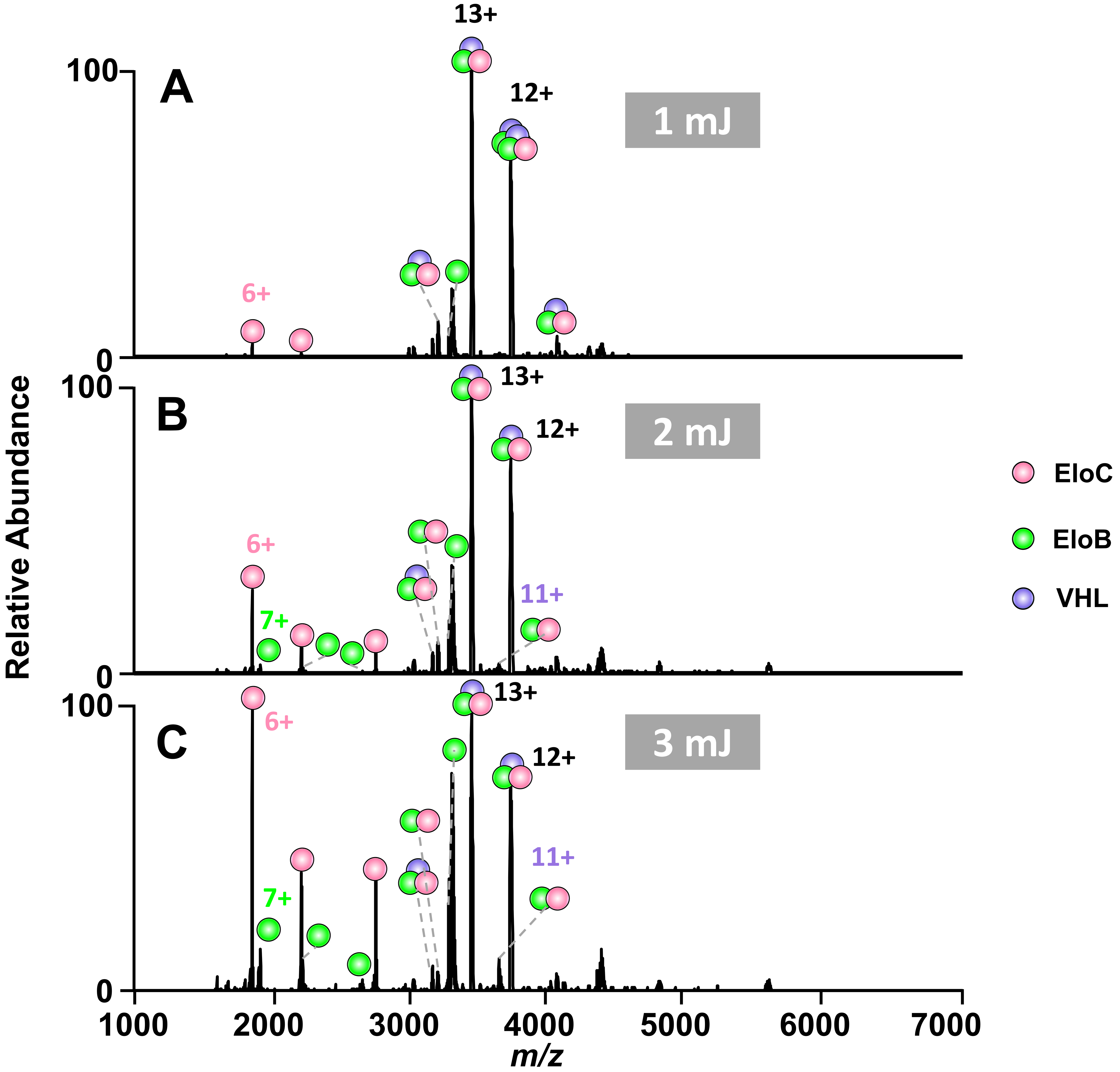

Figure S23. Dissociation behavior of the VCB E3 ligase complexes using UVPD. UVPD mass spectra of the VCB complexes (co-isolated 11+ to 14+ charge states) acquired using different UVPD energy (A) 1 mJ, (B) 2 mJ and (C) 3 mJ, revealing the progressive release of subunits EloB and EloC.

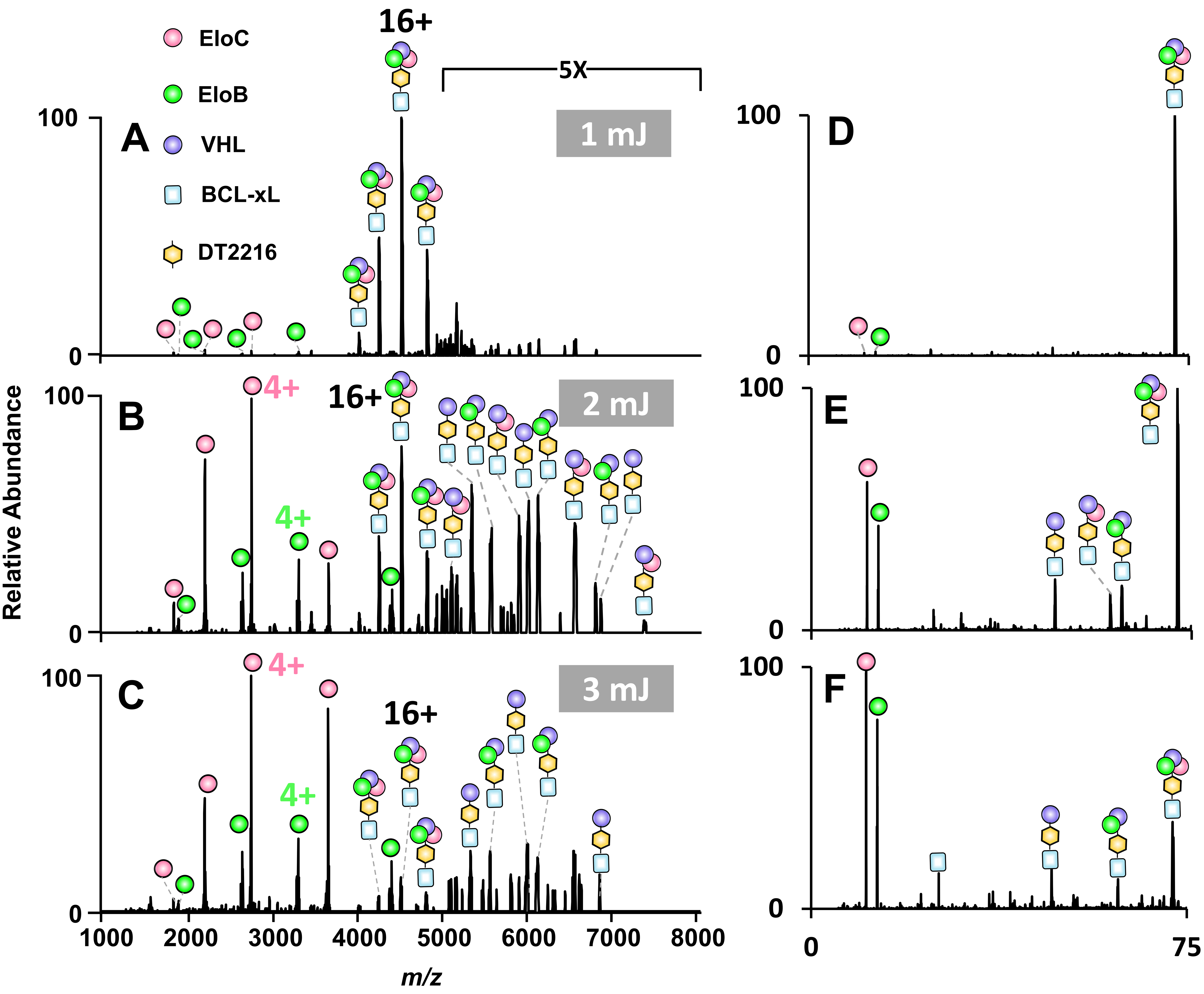

Figure S24. UVPD mass spectra of the ternary BCL-xL•DT2216•VCB complexes. A single mJ laser pulse was applied after co-isolation of ions in the *m/z* 4,000–5,000 range encompassing the 14+ to 18+ charge states. A) 1 mJ laser energy, B) 2 mJ laser energy, C) 3 mJ laser energy. (D-F) The corresponding deconvoluted mass spectra are shown on the right, confirming the preferential loss of EloB and EloC, while the DT2216 remains bound to the rest of the complex. These results suggest that UVPD leads to direct subunit dissociation with minimal disruption of the PROTAC-mediated interactions.

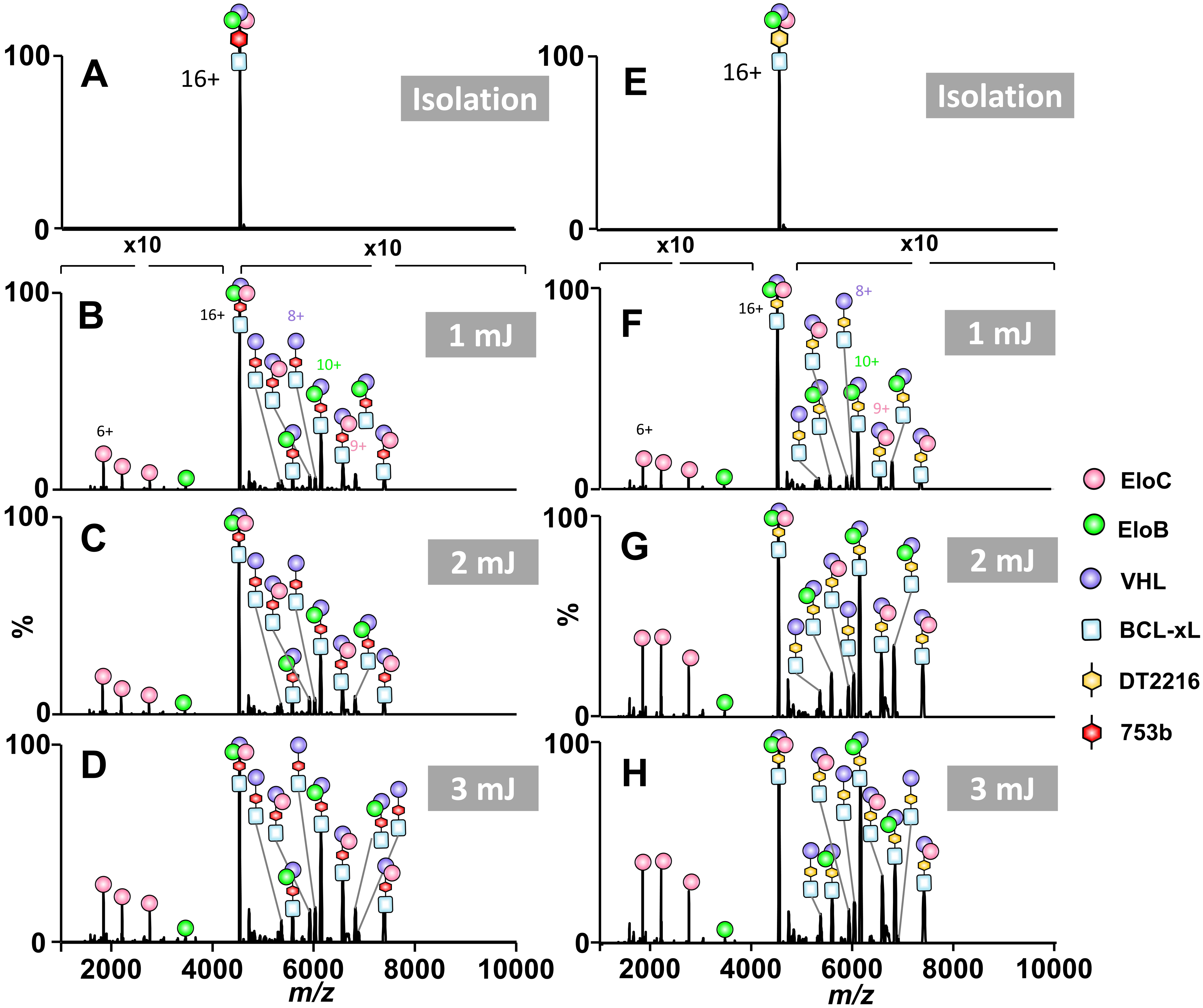

Figure S25. Isolation and UVPD of the ternary BCL-xL•PROTAC•VCB complexes (16+) for complexes generated from a solution containing 5 μM BCL-xL and 5 μM VCB incubated with 5 μM PROTAC (A-D) 753b or (E-F) DT2216. A) Isolation of BCL-xL•753b•VCB (16+) and UVPD using B) 1 mJ laser energy, C) 2 mJ laser energy, and D) 3 mJ laser energy. E) Isolation of BCL-xL•DT2216•VCB (16+) and UVPD using F) 1 mJ laser energy, G) 2 mJ laser energy, and H) 3 mJ laser energy. UVPD was performed using a single laser pulse.

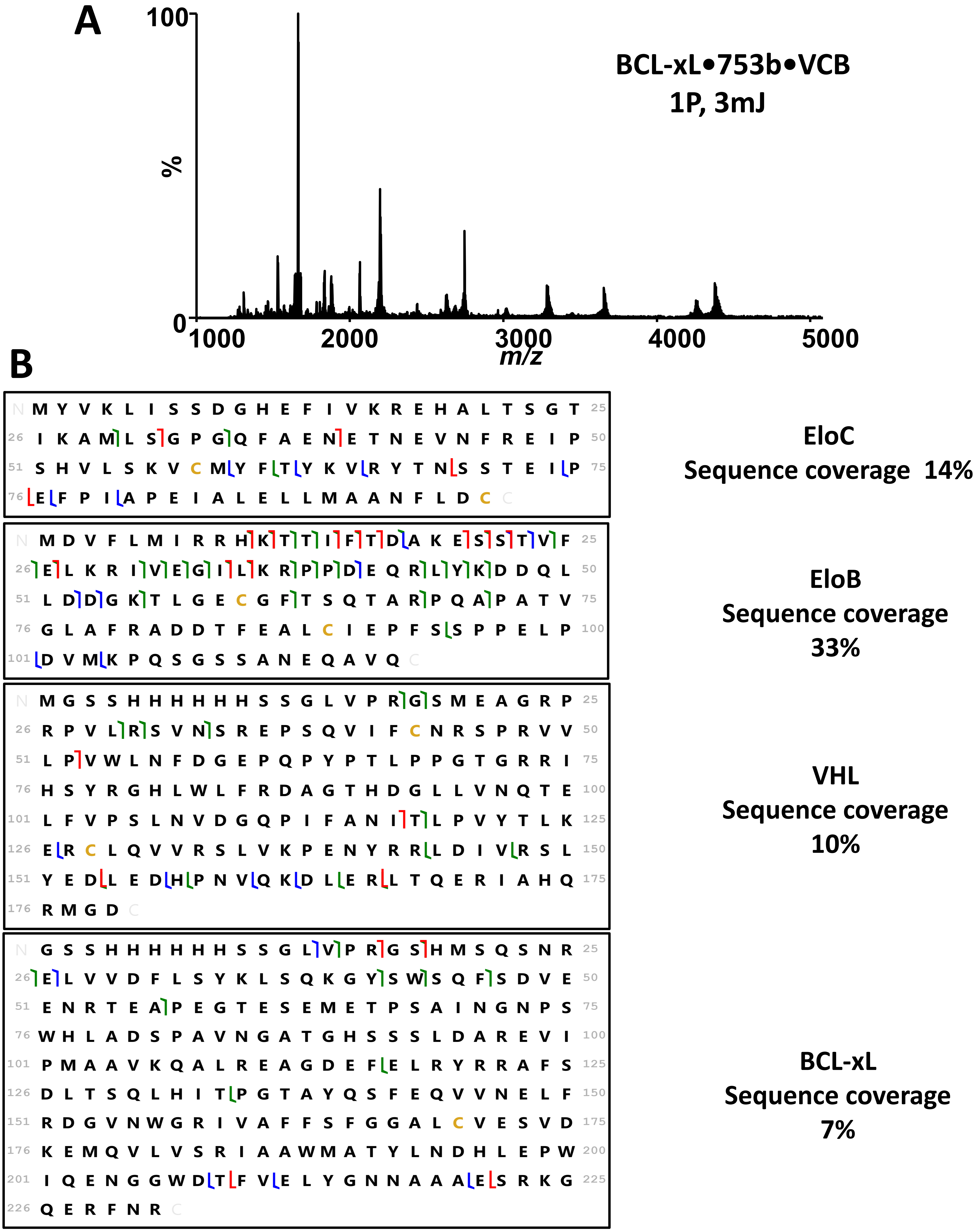

Figure S26. UVPD fragmentation of the BCL-xL•753b•VCB ternary complex in the 16+ charge state. (A) UVPD mass spectrum using single pulse and 3 mJ laser energy, 240 K resolution, and trapping gas setting = 2. The high-resolution, low-pressure conditions enhance detection of backbone-type fragment ions but also lead to a highly congested spectrum due to the large size of the ternary complex. (B) Sequence-coverage maps highlight the observed a, b, c, x, y, and z ions, confirming that the low-abundance peaks originate from backbone fragmentation rather than subcomplex dissociation. Low sequence coverage is obtained.

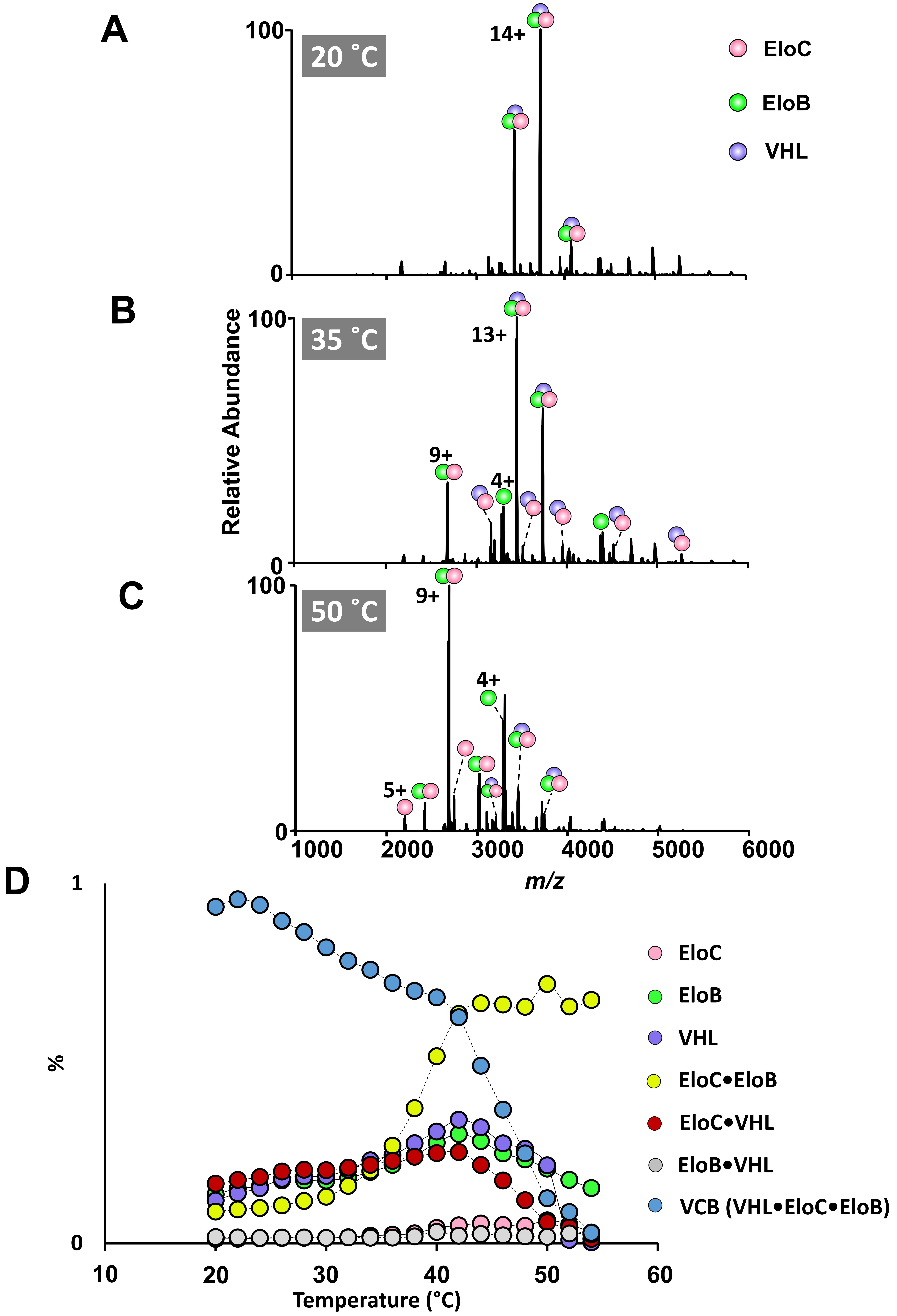

Figure S27. Thermal-induced dissociation of VCB complex using variable temperature-ESI-MS. The experiment was conducted using a solution containing 5 μM VCB in 100 mM ammonium acetate. The solution was gradually heated over a temperature range of 20°C to 55°C. Mass spectra were acquired at multiple temperature points including A) 20°C, B) 35°C and C) 50°C. (D) The relative abundances of key molecular species are plotted as a function of temperature, showing a stepwise dissociation pattern, with EloC•EloB released first, followed by the sequential dissociation of individual subunits.

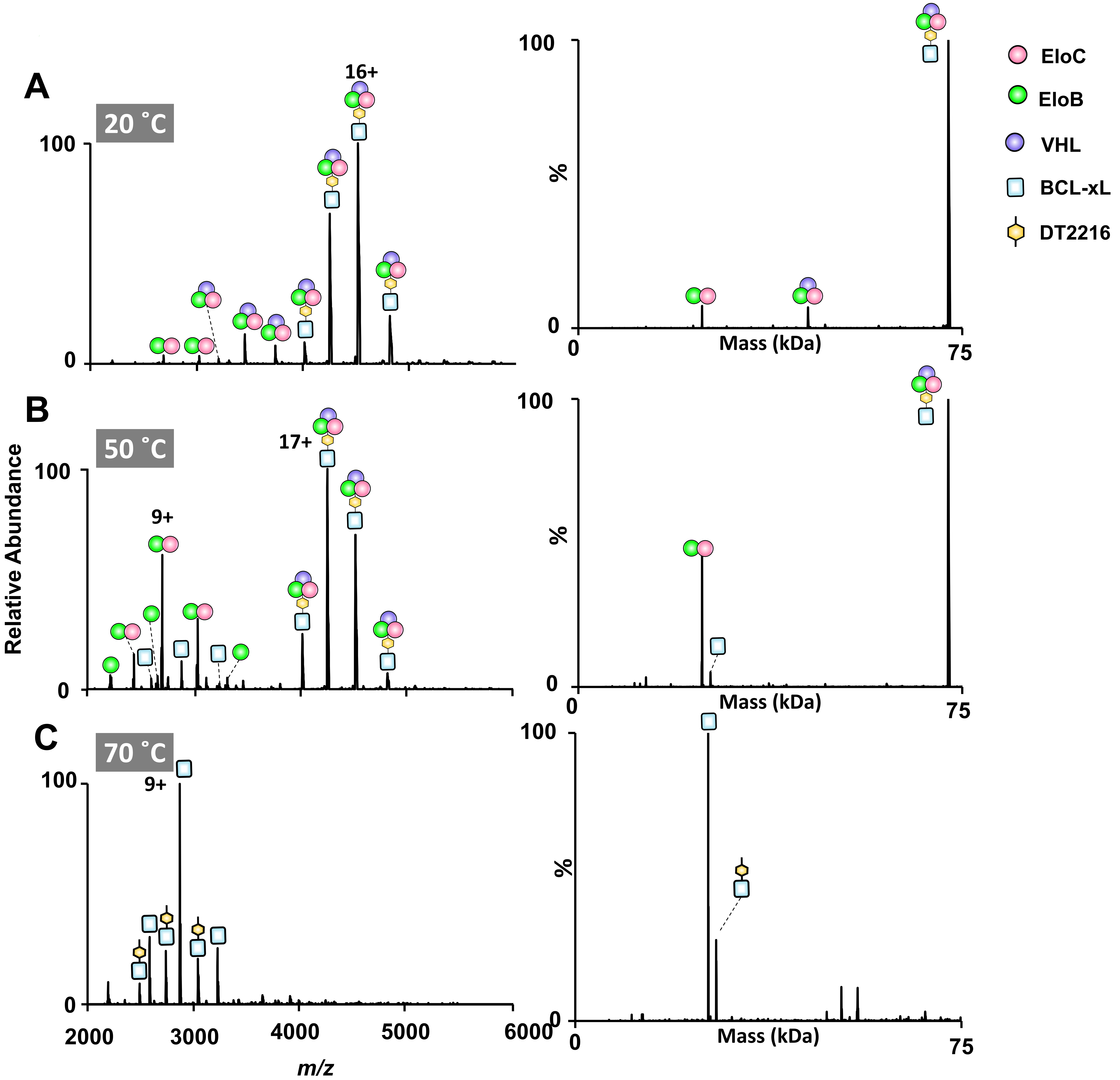

Figure S28. Evaluation of the thermal-induced stability of complexes containing DT2216 using variable temperature ESI-MS. The solution contained 5 μM BCL-xL, 5 μM VCB and 5 μM DT2216 in 100 mM ammonium acetate. The solution was gradually heated over a temperature range of 20°C to 70°C. Mass spectra were acquired at A) 20°C, B) 50°C, and C) 70°C. Deconvoluted mass spectra are shown on the right, confirming the sequential release of EloC•EloB followed by release of BCL-xL and disassembly of the binary BCL-xL•DT2216 complex.

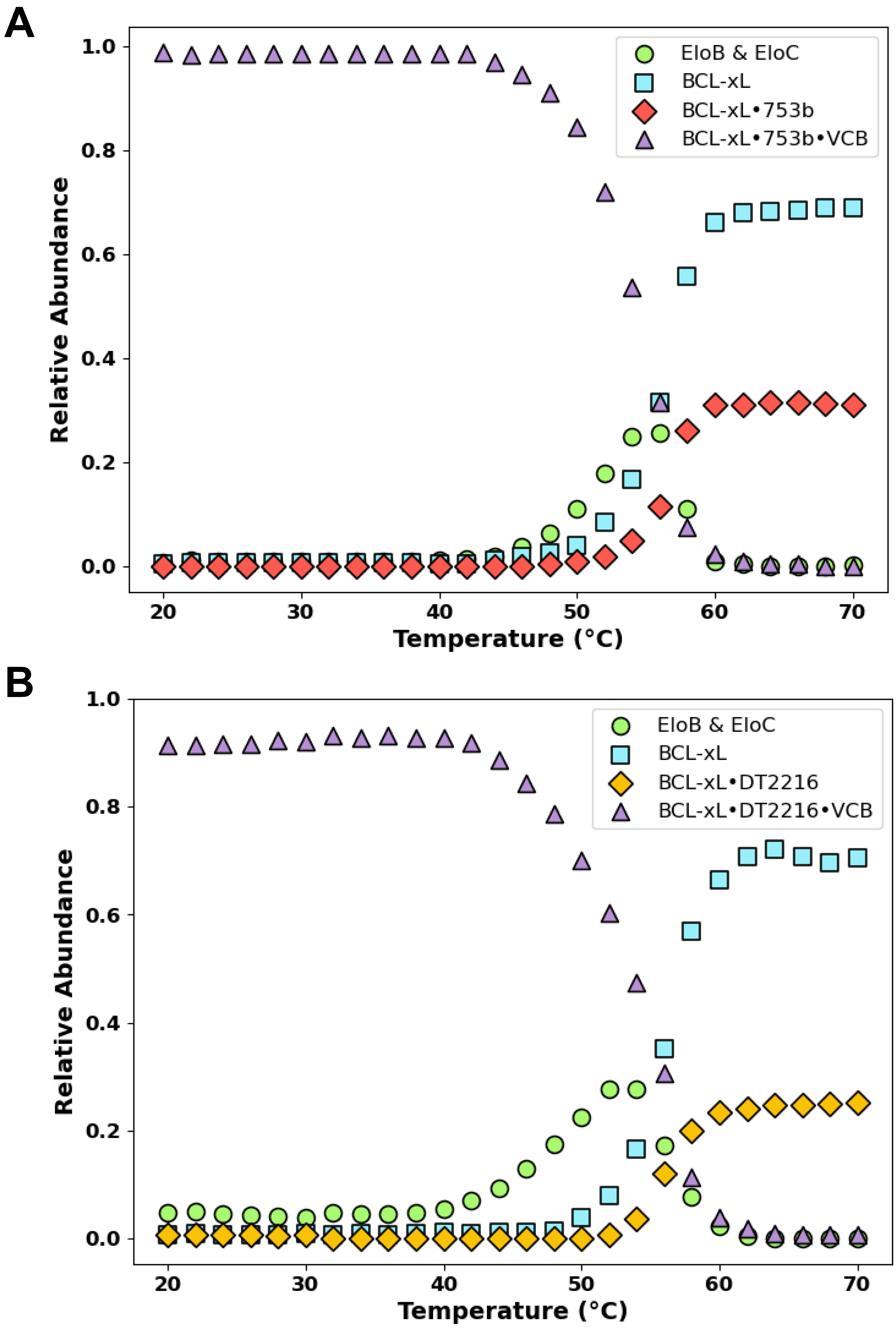

Figure S29. Temperature-dependent stability assay of different PROTAC complexes analyzed by variable temperature-ESI-MS. The relative abundances of key molecular species are plotted as a function of temperature for complexes in solutions containing A) BCL-xL, 753b, and VCB or (B) BCL-xL, DT2216 and VCB. The experiments were conducted using solutions containing 5 μM BCL-xL, 5 μM VCB and 5 μM PROTAC. There is a stepwise dissociation pattern, with EloB and EloC being released first, followed by the sequential dissociation of BCL-xL and BCL-xL•PROTAC binary complexes.

Plasmid cloning and protein expression

Different proteins were expressed and purified as previously described.[1,2] In brief, To enable co-expression of the VCB complex, a pACYC-Duet1 plasmid encoding full-length Elongin B and residues 17–112 of Elongin C was obtained (Addgene plasmid #110274). The VHL fragment (residues 54–213) was cloned into the pET28a vector containing an N-terminal 6×His tag followed by a thrombin cleavage site, enabling affinity purification and optional tag removal. For the expression of BCL-xL protein, codon-optimized gene fragments for BCL-xL (residues 2–212) was synthesized (Gene Universal, USA). The BCL-xL gene was inserted into a modified pSMT3.4 vector using NdeI and NotI restriction sites. This construct included an N-terminal SMT3 (SUMO) fusion tag to enhance solubility, which could be specifically cleaved using ULP1 protease. All constructs were verified by sequencing and transformed into BL21 (DE3) codon plus E. coli strain for protein expression.

Recombinant E. coli strains were cultured in LB medium at 37 °C with shaking until the optical density reach 1.5 at 600 nm. Protein expression was induced with 0.3 mM Isopropyl-β-D-thiogalactopyranoside, followed by overnight incubation at 18 °C. For VCB complex expression, co-transformation of the Elongin B/C plasmid and VHL construct was performed and induced under the same conditions. Cell pellets were harvested and resuspended in lysis buffer (50 mM Tris-HCl (pH 8.0), 300 mM NaCl, 5 mM β-mercaptoethanol, 5% glycerol, and 10 mM imidazole. Lysis was carried out via sonication followed by centrifugation at 35,000 × g for 30 minutes at 4 °C. The supernatant was applied to Ni-NTA agarose beads (Qiagen) for affinity purification. Proteins were released using elution buffer (50 mM Tris-HCl (pH 8.0), 200 mM NaCl, 300 mM imidazole, and 5 mM β-mercaptoethanol). BCL-xL purification tags were removed by treatment with SUMO ULP1 protease (1:1000 w/w) (1:100 w/w), through overnight incubation at 4 °C. The cleaved samples were then passed through Ni-NTA beads to remove non-cleaved protein, cleaved tag, and protease, followed by further purification using size-exclusion chromatography (Superdex 75). The VCB complex was purified with its His-tag intact and further processed using size-exclusion chromatography (Superdex 200) followed by anion-exchange purification (Mono Q 10/100). All proteins were concentrated to 4–8 mg/mL, aliquoted, flash-frozen in liquid nitrogen, and stored at −80 °C.

Synthesis of PROTACS:

The three PROTACs, DT2216,[3] 753b,[4] and PZ32652,[5] were synthesized as reported previously. Compound PZ32652 is a diastereomer of 753b, bearing a warhead derived from the enantiomer of ABT-263 (Navitoclax). According to the literature, the enantiomer of ABT-263 has significantly reduced binding affinity toward the target protein BCL-xL, although a measurable level of cellular activity is retained.[6] The synthesis of PZ32652 was accomplished by following the procedure reported for DT2216 and 753b, using the corresponding enantiomeric starting material.

Fluorescence polarization assay:

The binary binding of the test compounds to VCB complex was measured following the fluorescence polarization assay protocol reported in the literature.[7]

Authentication of materials.
The identities of all recombinant proteins, BCL-xL, and the VCB complex (VHL•EloB•EloC), were authenticated using a combination of native and denaturing MS, as described in **Supporting Information** (**Tables S1-S2, Figures S1-S3**). Although the observed experimental masses deviated from theoretical values, they were consistent between native and denaturing conditions. For example, the deconvoluted mass spectra for solutions contain BCL-xL yielded monoisotopic masses of 25,816 Da and 25,850 Da (native and denatured, respectively) compared to a theoretical mass of 26,809 Da (**Table S1)**. Similar modest mass discrepancies for experimental and theoretical masses **(Table S1**) were noted for EloC (10,900 Da vs. 10,832 Da), EloB (13,100 Da vs. 13,017 Da), and VHL (20,900 Da vs. 20,558 Da). These differences may reflect residual tags, minor truncations, post-translational modifications, or salt adducts, commonly encountered in recombinant expression systems (**Figures S1-S3**). Moreover, PROTAC identity (**Table S2**) and purity were similarly verified **(Figure S4**). To ensure that solvent systems required for PROTAC solubilization did not compromise protein structural integrity under native MS conditions, we evaluated the effects of commonly used organic solvents—DMSO and methanol—on the charge state distributions of BCL-xL and VCB proteins. Native mass spectra acquired in the presence of 10% DMSO showed a slight shift toward lower charge states, which is consistent with previous reports (**Figure S5**).[1] In contrast, 10% methanol did not cause any detectable change in charge state distribution compared to solvent-free conditions, suggesting minimal impact on native-like conformations. Based on these results, we selected methanol as the preferred solvent for preparation of PROTAC stock solutions in all subsequent experiments.

References

1) Sterling, H.J., Prell, J.A., Cassou, C.A., Williams,E.R., Protein Conformation and Supercharging with DMSO from Aqueous Solution. *Journal of the American Society for Mass Spectrometry* **2011,** 22(7) 1178-1186.

2) Claudia J. Diehl and Alessio Ciulli. Discovery of small molecule ligands for the von Hippel-Lindau (VHL) E3 ligase and their use as inhibitors and PROTAC degraders. *Chem. Soc. Rev*., **2022**, 51, 8216-8257. https://doi.org/10.1039/D2CS00387B

3) Khan, S.; Zhang, X.; Lv, D.; Zhang, Q.; He, Y.; Zhang, P.; Liu, X.; Thummuri, D.; Yuan, Y.; Wiegand, J. S.; Pei, J.; Zhang, W.; Sharma, A.; McCurdy, C. R.; Kuruvilla, V. M.; Baran, N.; Ferrando, A. A.; Kim, Y. mi; Rogojina, A.; Houghton, P. J.; Huang, G.; Hromas, R.; Konopleva, M.; Zheng, G.; Zhou, D. A Selective BCL-XL PROTAC Degrader Achieves Safe and Potent Antitumor Activity. *Nat Med* **2019**, 25 (12), 1938–1947. https://doi.org/https://doi.org/10.1038/s41591-019-0668-z.

4) Lv, D.; Pal, P.; Liu, X.; Jia, Y.; Thummuri, D.; Zhang, P.; Hu, W.; Pei, J.; Zhang, Q.; Zhou, S.; Khan, S.; Zhang, X.; Hua, N.; Yang, Q.; Arango, S.; Zhang, W.; Nayak, D.; Olsen, S. K.; Weintraub, S. T.; Hromas, R.; Konopleva, M.; Yuan, Y.; Zheng, G.; Zhou, D. Development of a BCL-xL and BCL-2 Dual Degrader with Improved Anti-Leukemic Activity,. *Nature Communications* **2021** 12:1 2021, 12 (1), 1–14. https://doi.org/10.1038/s41467-021-27210-x.

5) Pal, P.; Thummuri, D.; Lv, D.; Liu, X.; Zhang, P.; Hu, W.; Poddar, S. K.; Hua, N.; Khan, S.; Yuan, Y.; Zhang, X.; Zhou, D.; Zheng, G. Discovery of a Novel BCL-XLPROTAC Degrader with Enhanced BCL-2 Inhibition. *J Med Chem* **2021**, 64 (19), 14230–14246. https://doi.org/10.1021/ACS.JMEDCHEM.1C00517/SUPPL_FILE/JM1C00517_SI_002.CSV.

6) Tse, C.; Shoemaker, A. R.; Adickes, J.; Anderson, M. G.; Chen, J.; Jin, S.; Johnson, E. F.; Marsh, K. C.; Mitten, M. J.; Nimmer, P.; Roberts, L.; Tahir, S. K.; Xiao, Y.; Yang, X.; Zhang, H.; Fesik, S.; Rosenberg, S. H.; Elmore, S. W. ABT-263: A Potent and Orally Bioavailable Bcl-2 Family Inhibitor. *Cancer Res* **2008**, 68 (9), 3421-3428. https://doi.org/10.1158/0008-5472.CAN-07-5836.

7) Zoppi, V.; Hughes, S. J.; Maniaci, C.; Testa, A.; Gmaschitz, T.; Wieshofer, C.; Koegl, M.; Riching, K. M.; Daniels, D. L.; Spallarossa, A.; Ciulli, A. Iterative Design and Optimization of Initially Inactive Proteolysis Targeting Chimeras (PROTACs) Identify VZ185 as a Potent, Fast, and Selective von Hippel-Lindau (VHL) Based Dual Degrader Probe of BRD9 and BRD7. *J. Med. Chem*. **2019**, 62 (2), 699-726. https://doi.org/10.1021/acs.jmedchem.8b01413.
